# ASSET: Analysis of Sequences of Synchronous Events in Massively Parallel Spike Trains

**DOI:** 10.1371/journal.pcbi.1004939

**Published:** 2016-07-15

**Authors:** Emiliano Torre, Carlos Canova, Michael Denker, George Gerstein, Moritz Helias, Sonja Grün

**Affiliations:** 1 Institute of Neuroscience and Medicine (INM-6) and Institute for Advanced Simulation (IAS-6) and JARA BRAIN Institute I, Jülich Research Centre, Jülich, Germany; 2 Department of Neuroscience, University of Pennsylvania, Philadelphia, Pennsylvania, United States of America; 3 Department of Physics, RWTH Aachen University, Aachen, Germany; 4 Department of Biology, RWTH Aachen University, Aachen, Germany; Indiana University, UNITED STATES

## Abstract

With the ability to observe the activity from large numbers of neurons simultaneously using modern recording technologies, the chance to identify sub-networks involved in coordinated processing increases. Sequences of synchronous spike events (SSEs) constitute one type of such coordinated spiking that propagates activity in a temporally precise manner. The synfire chain was proposed as one potential model for such network processing. Previous work introduced a method for visualization of SSEs in massively parallel spike trains, based on an intersection matrix that contains in each entry the degree of overlap of active neurons in two corresponding time bins. Repeated SSEs are reflected in the matrix as diagonal structures of high overlap values. The method as such, however, leaves the task of identifying these diagonal structures to visual inspection rather than to a quantitative analysis. Here we present ASSET (Analysis of Sequences of Synchronous EvenTs), an improved, fully automated method which determines diagonal structures in the intersection matrix by a robust mathematical procedure. The method consists of a sequence of steps that i) assess which entries in the matrix potentially belong to a diagonal structure, ii) cluster these entries into individual diagonal structures and iii) determine the neurons composing the associated SSEs. We employ parallel point processes generated by stochastic simulations as test data to demonstrate the performance of the method under a wide range of realistic scenarios, including different types of non-stationarity of the spiking activity and different correlation structures. Finally, the ability of the method to discover SSEs is demonstrated on complex data from large network simulations with embedded synfire chains. Thus, ASSET represents an effective and efficient tool to analyze massively parallel spike data for temporal sequences of synchronous activity.

## Author Summary

Neurons in the cerebral cortex are highly interconnected. However, the mechanisms of coordinated processing in the neuronal network are not yet understood. Theoretical studies have proposed synchronized electrical impulses (spikes) propagating between groups of nerve cells as a basis of cortical processing. Indeed, animal studies provide experimental evidence that spike synchronization occurs in relation to behavior. However, the observation of sequences of synchronous activity has not been reported so far, presumably due to two fundamental problems. First, the long-standing lack of simultaneous recordings of large populations of neurons, which only recently were enabled by advances in recording technology. Second, the absence of proper tools required to find these activity patterns in such high-dimensional data. Addressing the second issue, we introduce here a fully automatized mathematical method that advances an existing visual approach to identify sequences of synchronous events in large data sets. We demonstrate the efficacy of our method on a range of simulated test data that capture the characteristics and variability of experimental data. Our tool will serve future studies in their search for spike time coordination at millisecond precision in the brain.

## Introduction

Synchronous input spikes to a receiving neuron are considered most effective in generating an output spike, as predicted by theoretical studies coining the term coincidence detector [1]. The argument rests on the premise that excitatory post-synaptic potentials (EPSPs) in the cortex are typically small in relation to the firing threshold, so that many EPSPs need to overlap to produce an output spike. Due to leak currents in the neuronal membrane, the firing threshold is reached with fewer spikes when these arrive synchronously at the post-synaptic neuron rather than sparsely, thus making the neuron behave like a coincidence detector. Experimental studies provide evidence for the existence of coincidence detectors (e.g., [2]) and relate them to various mechanisms of spike-timing dependent plasticity [3, 4] as well as to different encoding and decoding schemes [5, 6].

Cortical anatomy supports such considerations. Individual neurons receive synaptic connections from a large number of neurons (on the order of 10,000 in the human cortex, see e.g. [7]) and project to a similar number of other cells. Such a connectivity structure combined with suitable synaptic delays may produce spatio-temporal spike patterns, as proposed in [8–11]. A simple case is represented by a temporal sequence of synchronous events (SSE), each event consisting of synchronous spikes from a group of neurons.

The synfire chain model [8] is a neural network model that has been proposed to exhibit such activity through a suitable wiring of the neurons in the network in successive groups interconnected in a highly divergent and convergent manner. Each neuron of one group projects to several neurons of the next group, thus forming a chain structure. Under the assumption that the synaptic transmission delays from neurons of one group to neurons of the next group are identical, the synchronous stimulation of neurons in the first group leads to robust propagation of synchronous spiking activity through the chain [10] even in the presence of noise. The activation of a synfire chain would thus lead to the occurrence of an SSE.

In order to assess whether SSEs are indeed observed in the brain and have a functional role, the spiking activity of several neurons needs to be recorded simultaneously, analyzed for temporal correlation and related to behavior. Under the assumption that an SSE occurs sparsely in a given data set, for instance in relation to a specific behavior, pairwise correlations between neurons engaged in the activity are weak and will not be detected by means of pairwise correlation analyses. For this reason, an analysis method was proposed in [12] that directly searches for SSEs in massively parallel spike data.

**Fig 1:**
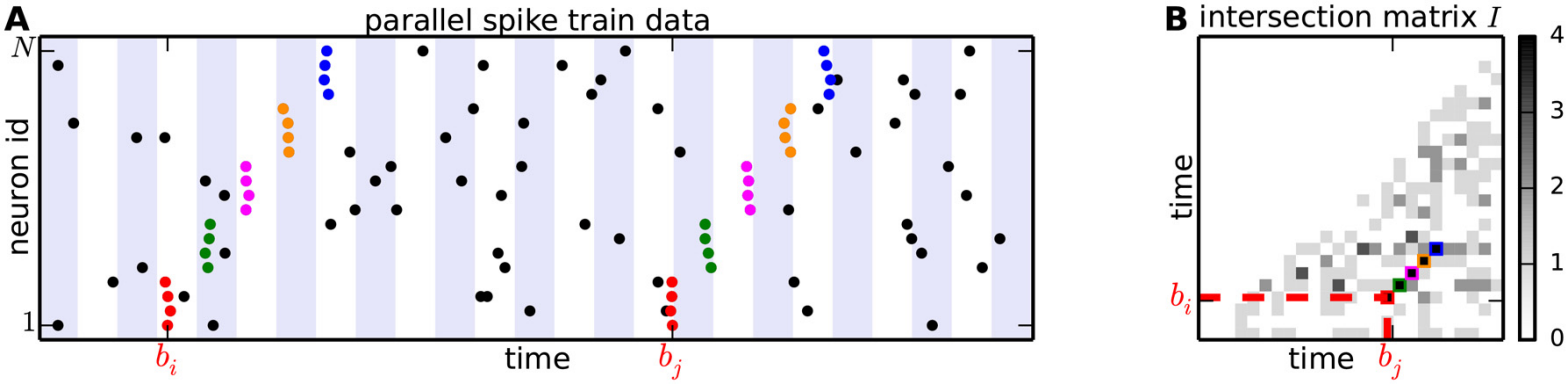
From spike trains to the intersection matrix. **(A)** Raster plot of parallel spike trains of multiple neurons (vertical axis) over time (horizontal axis). Dots in each row correspond to the spike times of one neuron. Time is discretized into adjacent bins (marked by white and blue shaded backgrounds) to define synchronous events. Synchronous spikes forming an SSE repeating twice are indicated by colored dots (one color per event). **(B)** Intersection matrix *I*. Each matrix entry *I*_*ij*_ (values encoded by gray levels) contains the degree of overlap of neurons active in time bins *b*_*i*_ and *b*_*j*_. Only the entries *I*_*ij*_ with *i* < *j* are shown due to the symmetry of the matrix.

The basic idea of the method presented in [12] is the following: after discretizing time in bins of a few ms (see Fig 1A), a synchronous event which repeats at two different time bins leads to a large number of neurons that are active in both bins. Building an intersection matrix *I* where each entry *I*_*ij*_ represents the number of neurons active in both bins *i* and *j*, a group of synchronous events occurring in bins *i* and *j* results in a large overlap *I*_*ij*_ compared to other entries of the matrix. An SSE which occurs twice produces in the matrix *I* a sequence of high-valued entries, which we name *diagonal structures* (DS), aligned parallel to the main diagonal (Fig 1B). A diagonal filter applied to the matrix *I* enhances the contrast between the DS and surrounding entries, mapping *I* into a filtered matrix *F*.

The method was calibrated using data from large-scale synfire chain network simulations. When the neurons of a full chain or a large portion of it are observed, the method reveals the associated DS in the intersection matrix. However, when only a few hundreds of neurons (comparable to the number of cells that can be recorded simultaneously *in vivo* with modern electrophysiological techniques) are randomly sampled from the full network, the DSs are less visible and less likely to be continuous, making it impossible to isolate them from the surrounding entries. Indeed, this method leaves the judgment of whether individual entries are large or small, as well as the grouping of proximal entries into DSs, to visual inspection. As a consequence, the results are prone to subjectiveness and the procedure is not open to automation.

In this paper we present a method, named ASSET (Analysis of Sequences of Synchronous EvenTs), which improves the approach proposed in [12] by providing a mathematical and fully automated detection of SSEs in parallel spike train data. The analysis features i) a statistical assessment of membership of individual entries of the intersection matrix to a DS, ii) a rigorous construction of individual DSs and associated SSEs by clustering, and iii) the reconstruction of the neuronal composition and occurrence times of each event in the found SSEs.

The manuscript is organized as follows. In “*Methods*” we derive a statistical assessment of the membership of matrix entries to a DS based on two statistical tests for the significance of each individual entry and the joint significance of an entry and its neighbors, respectively. Entries passing both tests are then grouped together into individual DSs depending on their reciprocal distance by a clustering procedure. Once the DSs are identified, it is readily possible to reconstruct their neuronal composition.

In “*Results*” we assess the performance and robustness of ASSET on various types of simulated data that replicate typical features of electrophysiological recordings. These include firing rate heterogeneity across neurons, variability over time and different types of correlation structure in the spiking activity. In addition, we demonstrate the ability of the ASSET analysis to find repeated SSE activity in simulated data generated by a neural network model of overlapping synfire chains as introduced in [12].

We conclude by discussing benefits and limitations of the ASSET method, open problems and future plans for the analysis of electrophysiological data.

## Methods

In this section we provide a formal definition of the intersection matrix as introduced in [12], introduce statistical tests to determine whether individual entries of the intersection matrix are part of any DS, and propose a clustering technique to group significant entries into the different DSs. After the last step, we can determine the occurrence times and neuronal composition of all events of the repeated SSEs associated to the DSs found. We then formally describe the stochastic models employed in “*Results*” to assess the performance of the method in a variety of scenarios.

### The intersection matrix

Our approach builds on the notion of intersection matrix defined in [12]. Here we introduce this concept mathematically and provide definitions. Given a set of *N* parallel spike trains observed in the time interval [0, *T*], synchronous spike events across neurons are determined by discretizing the time interval into *B* adjacent time bins *b*_1_, *b*_2_, …, *b*_*B*_ of identical width Δ = *T*/*B* (typically of a few ms), as illustrated in Fig 1A. Each set *S*_*i*_ of spikes falling in time bin *b*_*i*_ forms a synchronous event. The value |*S*_*i*_ ∩ *S*_*j*_| represents the number of neurons being active at both time bins *b*_*i*_ and *b*_*j*_. The *intersection matrix* *I* is defined by

$$\begin{array}{c} I_{ij}≔\left|S_{i}\cap S_{j}\right|\;\forall i,\;j=1,2,\ldots ,B. \end{array}$$

In this setting *I* is a symmetric matrix, whose main diagonal contains the population histogram, i.e. the time histogram of the number of neurons simultaneously active in each time bin. A synchronous spike event occurring at two bins *i* and *j*, e.g. as a result of the repeated activation of the same synfire chain, results in a larger value for *I*_*ij*_ compared to the chance level. A repeated SSE composed of *l*_SSE_ successive synchronous events, each of which occurs at time bins (*b*_*i*_*r*__, *b*_*j*_*r*__), *r* = 1, 2…, *l*_SSE_, determines a sequence of large-valued entries *I*_*i*_*r*_, *j*_*r*__ in *I*—which we term a *diagonal structure* (DS)—as sketched in Fig 1B.

To account for the variability of firing rates over time when comparing different entries of *I*, in [12] each entry of *I*_*ij*_ was normalized by the number of neurons active in each bin *b*_*i*_ and *b*_*j*_. After normalization, the entries take value 1 for a complete overlap and value 0 for no overlap. To enhance the contrast between high-valued entries belonging to the same diagonal structure and the surrounding entries, the matrix was further filtered by a linear kernel having an orientation parallel to the main diagonal. We take here a different approach, as outlined in the following section.

### Statistical significance of individual entries in the intersection matrix

To derive a measure of overlap that is independent of firing rates, we first need the probability mass function (pmf) *p*_*ij*_(⋅) of each individual entry *I*_*ij*_ in the intersection matrix, under the null hypothesis *H*_0_ that the spike trains under consideration are realizations of mutually independent Poisson processes. If the null hypothesis is rejected, i.e. if the observed value *ξ* taken by *I*_*ij*_ is too large to be interpreted as chance, we classify the overlap *S*_*i*_ ∩ *S*_*j*_ as a statistically significant repeated synchronous event. In “*Results*” we show that the statistics of the method are robust to deviations from Poissonianity as well as to the presence of various types of correlations other than repeated SSE activity.

Under the stated hypotheses, the distribution of *I*_*ij*_ is determined by the firing rate of each neuron *k* at the time bins *b*_*i*_ and *b*_*j*_. *I*_*ij*_ represents the (stochastic) number of neurons firing simultaneously in both bins and can thus be expressed as

$$\begin{array}{c} I_{ij}=\sum_{k=1}^{N}I_{ij}^{\left(k\right)}, \end{array}$$ (1)

where $I_{ij}^{\left(k\right)}$ is a Bernoulli random variable taking value 1 if neuron *k* fires in both bins *i* and *j*, and 0 otherwise. The probability parameter $p_{ij}^{\left(k\right)}$ of $I_{ij}^{\left(k\right)}$ is related to the local firing rate $\lambda_{i}^{\left(k\right)}$ of neuron *k* at bin *b*_*i*_ and $\lambda_{j}^{\left(k\right)}$ at bin *b*_*j*_ by

$$\begin{array}{c} p_{ij}^{\left(k\right)}=\left(1-e^{-\lambda_{i}^{\left(k\right)}\Delta }\right)\left(1-e^{-\lambda_{j}^{\left(k\right)}\Delta }\right) \end{array}$$

for each *i* ≠ *j*, where each factor $1-e^{-\lambda_{i}^{\left(k\right)}\Delta }$ is the probability for neuron *k* to emit at least one spike in the time bin *i*. The knowledge of the firing rate profiles of all neurons is therefore a prerequisite for the exact computation of the pmf *p*_*ij*_(⋅) of *I*_*ij*_, *i*, *j* = 1, …, *N*.

In light of Eq 1, *I*_*ij*_ is a Poisson Bernoulli random variable [13]. Its pmf *p*_*ij*_(⋅) is analytically given by

$$\begin{array}{c} p_{ij}\left(\xi \right)=\sum_{A\in P_{N;\xi }}\left\{\prod_{k\in A}p_{ij}^{\left(k\right)}\prod_{h\in A^{C}}\left(1-p_{ij}^{\left(h\right)}\right)\right\}, \end{array}$$ (2)

where $P_{N;\xi }$ is the family of all possible subsets of *ξ* elements that can be extracted from the set {1, …, *N*}, *A* is one such subset and *A*^*C*^ = {1, …, *N*}∖*A* is the complement of *A*. Thus *p*_*ij*_(*ξ*) is a summation of (Nξ) addenda, for a total of 2^*N*^ terms needed to compute *p*_*ij*_(⋅). The computation is feasible for small *N*, but soon becomes prohibitive as *N* increases beyond a few dozens, as in the applications to large parallel recordings we are interested in.

By use of Le Cam’s theorem [13], we approximate *p*_*ij*_(⋅) by a Poisson density function *p*_(*λ*)_ with rate parameter *λ* = ∑_*k*_ *λ*_*k*_

$$\begin{array}{c} p_{ij}\left(\xi \right)≃p_{\left(\lambda \right)}\left(\xi \right)=\frac{\lambda^{\xi }e^{-\lambda }}{\xi !}. \end{array}$$ (3)

**Fig 2:**
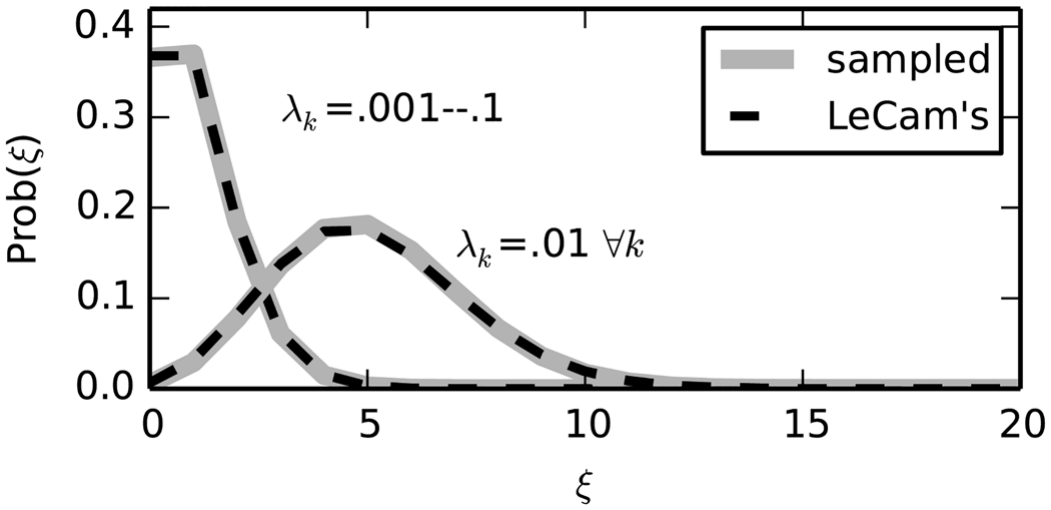
Le Cam’s approximation. The continuous gray curves show two Poisson Bernoulli distributions resulting from the sum of 100 Bernouilli random variables, having rates *λ*_*k*_ = 0.01 ∀*k* (right curve) and *λ*_*k*_ = 0.001, 0.002, …, 0.1 (left curve), respectively. The distributions are drawn as histograms from samples of size 10^8^ (calculating their exact analytical expression as in Eq 2 exact is computationally prohibitive). In both cases, Le Cam’s approximation given by a Poisson distribution with parameter *λ* = ∑_*k*_ *λ*_*k*_ (dashed lines) yields a good match (integrated absolute error <0.04).

The approximation error grows quadratically with the $p_{ij}^{\left(k\right)}$’s and stays low if the $p_{ij}^{\left(k\right)}$’s are sufficiently small, as shown in Fig 2.

**Fig 3:**
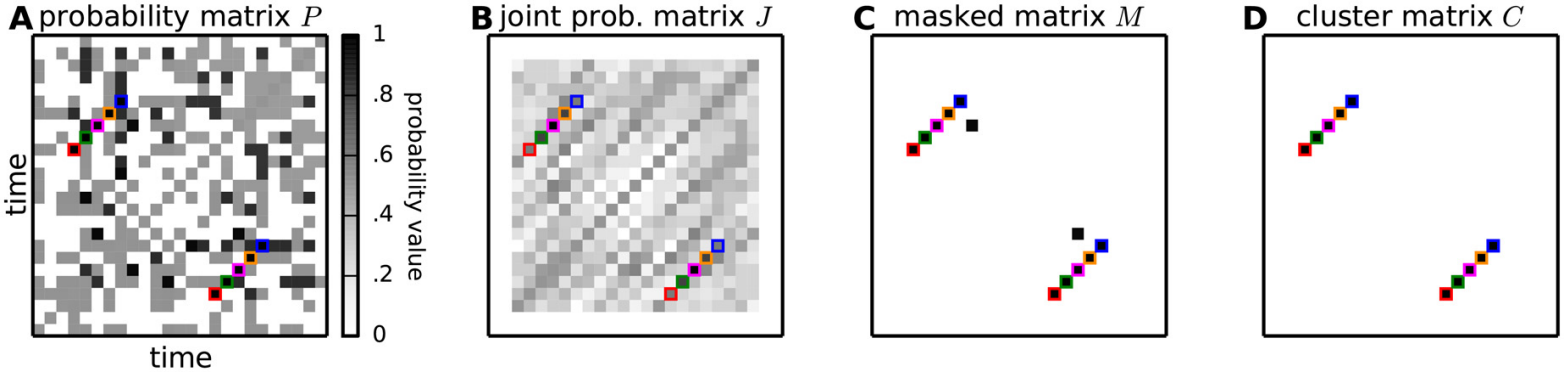
From spike trains to the cluster matrix. **(A)** Given parallel spike train data and their intersection matrix *I* as in Fig 1, for each entry *I*_*ij*_ its cumulative probability *P*_*ij*_ is calculated analytically under the null hypothesis $H_{0}$ that the spike trains are independent and marginally Poisson. **(B)** The *l* largest neighbors of *I*_*ij*_ in a rectangular area extending along the 45 degree direction are isolated by means of a kernel and their joint cumulative probability is assigned to the joint probability matrix *J* at position *J*_*ij*_. **(C)** Chosen a significance threshold *α*_1_ for the probability of individual entries (i.e. for entries *P*_*ij*_) and a significance threshold *α*_2_ for the joint probability of the neighbors of an entry (i.e. for entries *J*_*ij*_), each entry *I*_*ij*_ for which *P*_*ij*_ > *α*_1_ and *J*_*ij*_ > *α*_2_ is classified as statistically significant. Significant entries of *I* are retained in the binary masked matrix *M*_*ij*_, which takes value 1 at positions (*i*, *j*) where *I* is statistically significant and 0 elsewhere. **(D)** 1-valued entries in *M* falling close-by are clustered together (or discarded as isolated chance events) by means of a DBSCAN algorithm, which thus isolates diagonal structures.

Given the firing rate profile (i.e. the rate at each time bin) of each neuron, we can thus calculate the pmf *p*_*ij*_(⋅) and its cumulative distribution function (cdf) $P_{ij}\left(\cdot \right)≔\sum_{\xi <\cdot }p_{ij}\left(\xi \right)$, either exactly from Eq 2 or approximately relying on Le Cam’s approximation in Eq 3. Transforming each entry *I*_*ij*_ by its respective cdf $P_{ij}\left(\cdot \right)$, we map the observed overlaps *I*_*ij*_ to cumulative probabilities

$$\begin{array}{c} P_{ij}≔P_{ij}\left(I_{ij}\right)=\sum_{\xi <I_{ij}}\frac{\lambda^{\xi }e^{-\lambda }}{\xi !}, \end{array}$$ (4)

obtaining the *probability matrix* *P* ≔ (*P*_*ij*_)_*ij*_, as illustrated in Fig 3A.

If the null hypothesis holds, then $P_{ij}\left(\cdot \right)$ is the true probability distribution of the amount of overlap between bins *b*_*i*_ and *b*_*j*_, and *I*_*ij*_ is a realization from that probability distribution. If so, $P_{ij}:=P_{ij}\left(I_{ij}\right)$ takes *N* + 1 values *x* ∈ [0, 1) (as many as the intersection values from 0 to *N*), and its cdf is the identity function over the domain of this set of values: Pr(*P*_*ij*_ < *x*) = *x* for any *x*. If *P*_*ij*_ is large (close to 1), the null hypothesis is rejected in favor of the alternative hypothesis that the observed overlap reflects active synchronization between the involved neurons at time bins *b*_*i*_ and *b*_*j*_. After setting a significance threshold *α*_1_ (e.g. *α*_1_ = 0.99), we classify all entries *I*_*ij*_ for which *P*_*ij*_ > *α*_1_ as statistically significant, along with the associated repeated synchronous events.

Note that the sum in Eq 4 includes only values of *ξ* strictly lower than *I*_*ij*_. This choice, which assigns the observed value *I*_*ij*_ to the critical region of the hypothesis test, ensures that 1 − *P*_*ij*_ retains the property of a p-value, namely that Pr(1 − *P*_*ij*_ ≤ *y*) = *y* for each *y*. 1 − *P*_*ij*_ reflects the probability, computed under the null hypothesis, that *I*_*ij*_ would take a value equal to or exceeding the observed value. We reject the null hypothesis if this probability is lower than 1 − *α*_1_ = 0.01.

### Joint significance of neighbors of *I*_*ij*_

A DS resulting from a repeated SSE, as sketched in Fig 1B, differs from the surrounding entries of the intersection matrix *I* due to not just one, but a sequence of entries with large values aligned around a diagonal. We devise a statistical test that exploits this feature to detect DSs in the intersection matrix. In the previous section we have already derived the probability matrix *P* as a transformation of *I* that normalizes raw intersection values by the local neuronal firing rates. We can now look for DSs in *P* rather than in *I*.

#### Neighbors extracted by rectangular kernel

A visual technique to enhance DSs in the intersection matrix was proposed in [12], consisting in averaging each entry *I*_*ij*_ in the matrix (previously normalized by estimates of the instantaneous rates) with surrounding entries falling in the same diagonal, and thus building a filtered version *F* of the normalized intersection matrix. *F*_*ij*_ is large if the neighbors *I*_*i*+*h*, *j*+*h*_ of *I*_*ij*_ are jointly large, and small otherwise.

**Fig 4:**
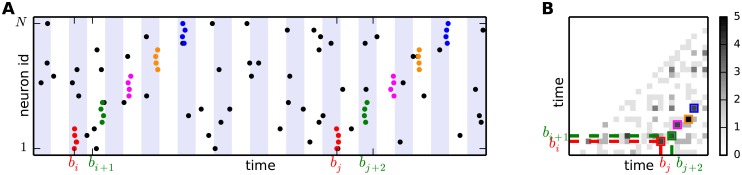
Origin of holes and wiggliness of a DS. Spiking activity containing a repeated SSE **(A)** and associated intersection matrix **(B)**. The SSE comprises 5 successive events. If two successive events of the SSE do not fall into adjacent bins (e.g. the second and third events in the first SSE repetition) a hole appears in the associated DS. If the distance (in number of bins) of two consecutive events is not identical for the two SSE repetitions, the corresponding entries belong to different off-diagonals of the intersection matrix.

Inspired by this approach, we aim at mapping the matrix *I* into a joint probability matrix *J* whose entries *J*_*ij*_ reflect the joint probability of entries surrounding *I*_*ij*_. We first note that the DS does not have to be composed of adjacent entries in *I*, and that these entries do not necessarily lie on the same off-diagonal. For instance, if any *r*-th entry of the DS, *r* ∈ {1, 2, …, *l*_SSE_}, is such that *i*_*r*_ > *i*_*r* − 1_+1 and/or *j*_*r*_ > *j*_*r* − 1_+1, then (*i*_*r* − 1_, *j*_*r* − 1_) and (*i*_*r*_, *j*_*r*_) will not be adjacent. If *i*_*r*_ − *i*_*r* − 1_ ≠ *j*_*r*_ − *j*_*r* − 1_, then (*i*_*r* − 1_, *j*_*r* − 1_) and (*i*_*r*_, *j*_*r*_) will not lie on the same diagonal, as sketched in Fig 4. The number of parallel diagonals spanned by the DS, or its “wiggliness”, is given by

$$\begin{array}{c} w_{\text{DS}}:=\max_{r}\left\{\Delta i_{r}-\Delta j_{r}\right\}-\min_{r}\left\{\Delta i_{r}-\Delta j_{r}\right\}+1, \end{array}$$

where Δ*i*_*r*_: = *i*_*r*_ − *i*_1_ and Δ*j*_*r*_: = *j*_*r*_ − *j*_1_ are the lags (in number of bins) of the *r*-th synchronous event from the first one, for the first and second occurrence of the SSE, respectively.

To select the entries around *I*_*ij*_ for which to calculate the joint probability, we center a rectangular kernel of length *l*_K_ and width *w*_K_ around *I*_*ij*_, aligned along the diagonal *I*_*ij*_ belongs to. If *I*_*ij*_ belongs to a DS, the kernel should optimally cover all the entries of the same DS. A rectangular kernel having a width *w*_K_ > 1 allows capturing wiggly DSs, contrarily to a linear kernel. We call the set of matrix entries covered by the kernel the *neighborhood* of *I*_*ij*_. If the intersection matrix between two different data segments is calculated, the neighborhood covers $n=l_{K}\cdot w_{K}-\lfloor \frac{w_{K}}{2}\rfloor (\lfloor \frac{w_{K}}{2}\rfloor +1)$ entries in total. However, for the intersection matrix between one and the same stretch of data, the main diagonal contains the population histogram and is therefore excluded from the analysis, as is the lower triangular matrix *i* > *j* that contains redundant information due to the symmetry of the matrix in this case. The total number *n* of entries considered in this case is re-calculated accordingly. A similar argument holds for entries *I*_*ij*_ close to the edge of the matrix.

**Fig 5:**
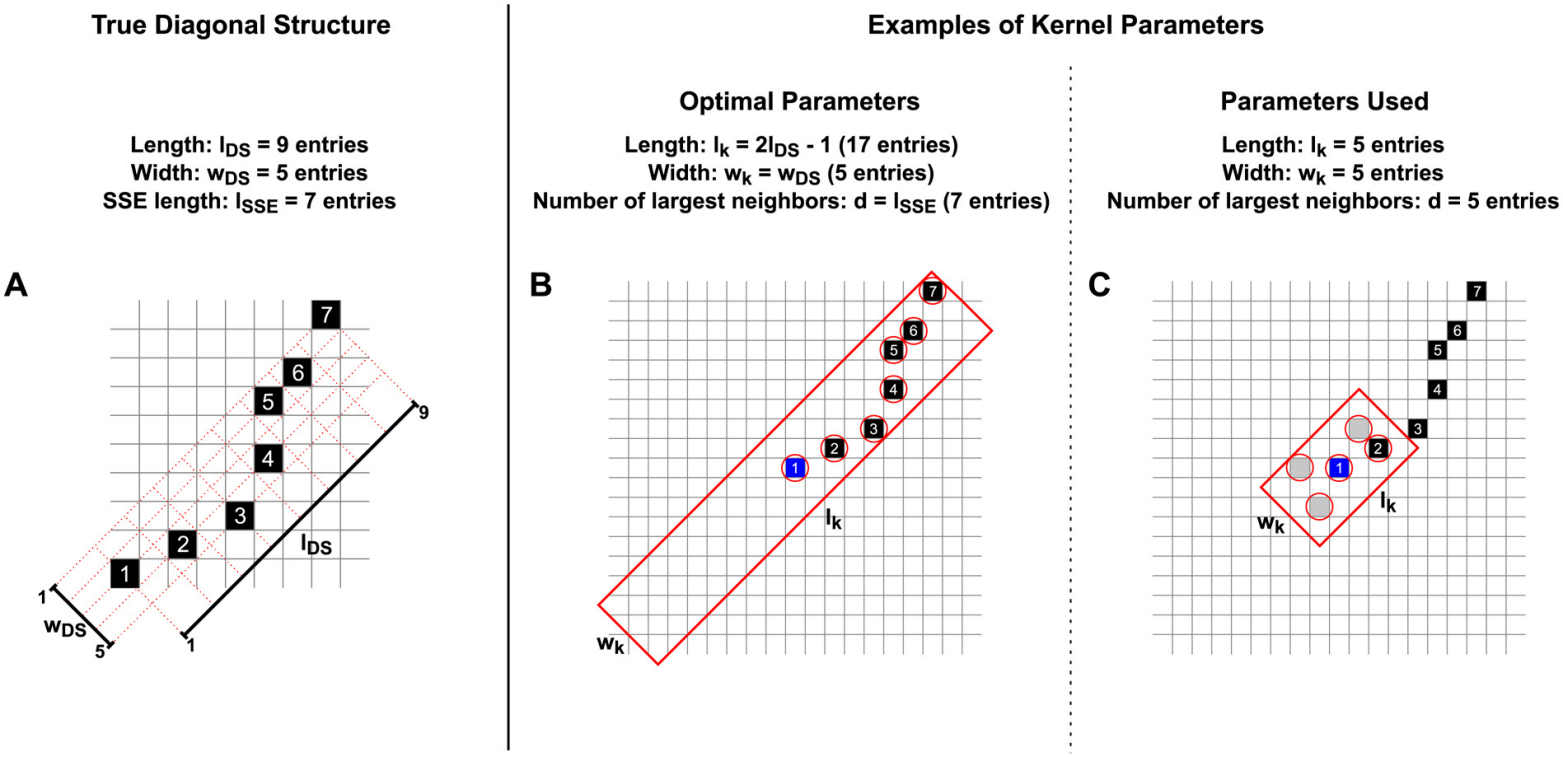
Kernel covering a diagonal structure. (A) Illustration of a DS in the intersection matrix composed of *l*_SSE_ = 7 entries, numbered 1 to 7. The DS has length *l*_DS_ = 9 (larger than *l*_SSE_, because some entries are separated by holes) and wiggliness parameter *w*_*DS*_ = 5 (because the entries are spread over 5 different diagonals). **(B)** Kernel centered around the first DS entry (in blue) with optimal parameters *l*_K_ = 2 · *l*_DS_ − 1 = 17 and *w*_*K*_ = *w*_DS_ = 5, ensuring coverage of the full DS. The largest *d* = 7 entries inside the kernel (circled) are considered for joint significance, in this case corresponding to the 7 DS entries. **(C)** Kernel with sub-optimal parameters *l*_*K*_ = 5, *w*_K_ = 5 and *d* = 5 (as employed in the validation, see “Results”), centered around the first DS entry. The kernel covers only 2 DS entries, and additional background entries are considered for joint significance.

Even when *I*_*ij*_ belongs to a DS fully covered by the kernel, only some of the *n* neighbor entries belong to the DS. For this reason we select only the *d* largest (i.e. most significant) entries in the neighborhood of *P*_*ij*_ and evaluate their joint statistical significance. The parameters *l*_K_, *w*_K_ and *d* reflect the putative length *l*_DS_ of the DS, its wiggliness *w*_DS_ and the length *l*_SSE_ of the SSE, respectively. As a rule, we set *l*_K_ and *w*_K_ odd so that the kernel is symmetric around *I*_*ij*_. The optimal choice of the parameters would be *l*_K_ = 2*l*_DS_ − 1 and *w*_K_ = *w*_DS_, such that the kernel fully covers the DS also when placed at one end of the DS itself (see Fig 5B). If the kernel length *l*_K_ is larger than this optimal value, the kernel would cover all entries forming the DS plus additional background entries. However, as only the *d* largest entries are considered for the joint significance, the background entries would not lower the joint significance and thus would not reduce the power of the test. Conversely, a smaller value for *l*_K_ results in a kernel that covers only part of the DS, thus yielding decreased joint probability values in the matrix *J* (see Fig 5C). However, the method tolerates sub-optimal values for *l*_K_ because the joint significance is already very high when the kernel covers just a few (e.g. 2 or 3) entries. This is indeed the case we investigated in our validation (see “Results”), where we set the filter length to half of the optimal length. These considerations show that the performance of the method is robust with respect to departures of the value of *l_K_* from the optimal choice.

The kernel width *w*_K_ reflects the putative wiggliness *w*_*DS*_ of the DS, and *w*_*K*_ = 5 corresponds to a value which accounts for highly wiggly DSs, as the one illustrated in Fig 5. Indeed, the events of the SSE are captured even if their delay from the first event changes between the two repetitions of the SSE by up to ±2 time bins. We set *w*_*K*_ = 5 in our validation (see “Results”) in order to capture this bad case scenario. This value was larger than the real wiggliness of the DSs we actually generated in our test data, *w*_DS_ ∈ {1, 2}, so that the kernel spanned more diagonals than the DS. The performance was nevertheless close to maximal: as for the parameter *l*_K_, the method tolerates excessively large values of *w*_K_ because only the *d* largest entries falling into the kernel are considered.

Finally, setting *d* = *l*_SSE_ would ensure that only the *l*_SSE_ largest entries (likely to be those forming the DS, if any) are considered, and not all the *n* entries covered by the kernel. Deviations from this optimal value amount to include additional or fewer entries than those forming the DS. In “*Results*” we show that, however, non optimal values are also tolerated. Indeed, as soon as 2 to 3 DS entries are considered among the *d* = 5 that are selected to determine the joint significance in our validation, the statistical test crosses significance threshold in almost all the cases (FN rate close to 0).

#### Significance of the *d* largest kernel entries

Under the null hypothesis *H*_0_ that the spike trains are independent and Poisson, we calculate the joint cumulative distribution of those *d* neighbors of *I*_*ij*_ whose corresponding cumulative probabilities *P*^(1)^, *P*^(2)^, …, *P*^(*d*)^ (already calculated and stored in the probability matrix *P*) are the largest among the *n* neighbors covered by the kernel. We consider *P*^(1)^, *P*^(2)^, …, *P*^(*d*)^, of which we aim to evaluate the joint significance, as independent samples from the same cumulative probability distribution *F*(*x*): = Pr(*P*^(*k*)^ < *x*) = *x*. Indeed, under *H*_0_ each entry *P*_*ij*_ is the cumulative probability of the corresponding intersection value, and its distribution is therefore the identity function over the discrete domain of the possible intersection values from 0 to *N*. These entries are not exactly independent if they share an index, e.g. (*i*, *j*_1_) and (*i*, *j*_2_). The approximation as independent samples, however, yields high performance, as shown in the validation (see “Results”).

For a sample (*X*_1_, *X*_2_, …, *X*_*n*_) of *n* independent realizations extracted from a probability distribution *F* defined on the interval [0, 1], the joint survival function $\bar{F}$ of the *d* largest order statistics *X*^(*n* − *d*+1)^ < *X*^(*n* − *d*+2)^ < … < *X*^(*n*)^ is defined by

$$\begin{array}{c} {\bar{F}}_{n-d+1,\ldots ,n}\left(x_{1},x_{2},\ldots ,x_{d}\right)≔Pr\left(X^{\left(n-d+1\right)}\geq x_{1},\;X^{\left(n-d+2\right)}\geq x_{2},\ldots ,\;X^{\left(n\right)}\geq x_{d}\right) \end{array}$$

and represents the probability that at least *d* samples are larger than or equal to *x*_1_, at least *d* − 1 samples are larger than or equal to *x*_2_, …, and at least 1 sample is larger than or equal to *x*_*d*_.

Since the *x*_1_ ≤ … ≤ *x*_*d*_ are ordered, the distribution vanishes if at least *d* samples are not ≥*x*_1_. Hence the number *i*_1_ of samples ≥*x*_1_ can take values *i*_1_ = *d*, *d*+1, …, *n* and the remaining *n* − *i*_1_ samples are below *x*_1_. Of the *i*_1_ samples ≥*x*_1_, there must be at least *d* − 1 samples ≥*x*_2_. Of the *i*_2_ samples ≥*x*_2_, *i*_2_ = *d* − 1, *d*, …, *i*_1_, there must be at least *d* − 2 samples ≥*x*_3_ and so on. Continuing this reasoning we arrive at the expression

$$\begin{array}{c} {\bar{F}}_{n-d+1}{}_{,\ldots ,n}\left(x_{1},x_{2},\ldots ,x_{d}\right)\\ \;=\sum_{i_{1}=d}^{n}\;\sum_{i_{2}=d-1}^{i_{1}}\ldots \sum_{i_{d}=1}^{i_{d-1}}\text{Pr}\left(\text{exactly}\;i_{k}\;\text{X}’\text{s}\;\text{are}\;\geq x_{k},\;k=0,1,\ldots ,d\right)\\ \;=\sum_{i_{1}=d}^{n}\;\sum_{i_{2}=d-1}^{i_{1}}\ldots \sum_{i_{d}=1}^{i_{d-1}}\text{Pr}\left(\text{exactly}\;i_{k}-i_{k+1}\;\text{X}’\text{s}\;\text{are}\;\text{in}\;[x_{k},\;x_{k+1}),\;k=0,1,\ldots ,d\right)\\ \;=\sum_{i_{1}=d}^{n}\;\sum_{i_{2}=d-1}^{i_{1}}\ldots \sum_{i_{d}=1}^{i_{d-1}}n!\prod_{k=0}^{d}\frac{{\left[F\left(x_{k+1}\right)-F\left(x_{k}\right)\right]}^{i_{k}-i_{k+1}}}{(i_{k}-i_{k+1})!}, \end{array}$$

for any *x*_1_ ≤ *x*_2_ ≤ … ≤ *x*_*d*_, and 0 otherwise, where *x*_0_ = 0, *x*_*d*+1_ = 1, *i*_0_ = *n* and *i*_*d*+1_ = 0.

Substituting to *F*(*x*_*r*_) → *P*^(*r*)^ and denoting for simplicity ${\bar{F}}_{n-d+1,\ldots ,n}$ by $\bar{F}$ yields

$$\begin{array}{c} \bar{F}\left(P^{\left(1\right)},P^{\left(2\right)},\ldots ,P^{\left(d\right)}\right)=n!\sum_{i_{1}=d}^{n}\;\sum_{i_{2}=d-1}^{i_{1}}\ldots \sum_{i_{d}=1}^{i_{d-1}}\;\prod_{k=0}^{d}\frac{{\left(P^{\left(k+1\right)}-P^{\left(k\right)}\right)}^{i_{k}-i_{k+1}}}{(i_{k}-i_{k+1})!}, \end{array}$$ (5)

which represents the upper-tail probability of the joint event

$$\begin{array}{c} \left(X^{\left(n-d+1\right)}\geq P^{\left(1\right)}\right)\cap \;\left(X^{\left(n-d+2\right)}\geq P^{\left(2\right)}\right)\cap \;\ldots \cap \;\left(X^{\left(n\right)}\geq P^{\left(d\right)}\right) \end{array}$$

under *H*_0_.

The upper-tail probability is small (that is, reflects high significance) either if *P*^(1)^, *P*^(2)^, …, *P*^(*d*)^ are jointly large (for instance, all >0.99) or if just one of them (e.g. the largest, *P*^(*d*)^) is extremely large (e.g. 0.99999999). The first scenario indicates the presence of a possible DS, while the second corresponds to the presence of an isolated entry which is individually so large (tail probability very close to 1) that its joint significance with neighboring entries taking values at chance level still crosses the significance threshold *α*_2_. To avoid the second scenario we impose an upper bound on the values of *P* by replacing each *P*^(*r*)^, *r* = 1, …, *d*, with $P_{*}^{\left(r\right)}=\min\left(P^{\left(r\right)},\;p_{\max}\right)$ (e.g. *p*_max_ = 0.999) to obtain

$$\begin{array}{c} \bar{F}\left(P^{\left(1\right)},P^{\left(2\right)},\ldots ,P^{\left(d\right)}\right)=n!\sum_{i_{1}=d}^{n}\;\sum_{i_{2}=d-1}^{i_{1}}\ldots \sum_{i_{d}=1}^{i_{d-1}}\prod_{k=0}^{d}\frac{{\left(P_{*}^{\left(k+1\right)}-P_{*}^{\left(k\right)}\right)}^{i_{k}-i_{k+1}}}{(i_{k}-i_{k+1})!}. \end{array}$$ (6)

By doing so, statistically highly significant values of $\bar{F}$ are possible only in the presence of jointly large neighbors of *P*_*ij*_, and not, as in the original expression Eq 5, when isolated entries take extremely high values while neighboring entries are at chance level.

The complementary function $1-\bar{F}\left(\cdot \right)$ returns the probability of *not* having the joint event, and can be used to map the probability matrix *P* into a *joint probability matrix* *J*

$$\begin{array}{c} P_{ij}\to J_{ij}=1-\bar{F}\left(P^{\left(1\right)},\;P^{\left(2\right)},\ldots ,\;P^{\left(d\right)}\right). \end{array}$$ (7)

Fig 3B illustrates the joint probability matrix *J* derived from the probability matrix *P* in panel A.

After setting a significance threshold *α*_2_ (e.g. *α*_2_ = 0.99999), we classify entries *I*_*ij*_ in the raw intersection matrix (Fig 1B) as having significantly jointly large neighbors if *J*_*ij*_ > *α*_2_.

### Clustering entries of *I* into DSs

Each entry *I*_*ij*_ in the raw intersection matrix is tested for its individual significance and for the joint significance of its neighbors. If both tests pass, i.e. if *P*_*ij*_ > *α*_1_ and *J*_*ij*_ > *α*_2_, the entry is classified as belonging to one DS. Such entries are collected in a binary *masked matrix* *M* that takes value 1 if both tests pass, and 0 otherwise,

$$\begin{array}{c} M_{ij}≔1_{\left\{I_{ij}>\alpha 1\right\}}\cdot 1_{\left\{J_{ij}>\alpha_{2}\right\}}, \end{array}$$ (8)

as illustrated in Fig 3C. It remains to be established which entries belong together to the same DS. Intuitively, entries in the masked matrix that take value 1 belong to the same DS if close-by, and to different DSs if far apart. The masked matrix in Fig 3C shows for instance two clearly separated DSs.

A suitable notion of “distance” for matrix entries should make entries falling in the same diagonal, i.e. aligned along the natural direction of a DS, closer together than entries aligned along the anti-diagonal. We introduce the following elliptic distance between any two matrix entries (*i*_1_, *j*_1_) and (*i*_2_, *j*_2_):

$$\begin{array}{c} d_{\rho }\left(\left(i_{1},j_{1}\right),\left(i_{2},j_{2}\right)\right)≔\left[1+\left(\rho -1\right)\cdot \sin\left(\left|\theta -\frac{\pi }{4}\right|\right)\right]\cdot \sqrt{{\left(i_{2}-i_{1}\right)}^{2}+{\left(j_{2}-j_{1}\right)}^{2}}/\sqrt{2}, \end{array}$$

where $\theta =\arctan(\frac{j_{2}-j_{1}}{i_{2}-i_{1}})$ is the angular coefficient of the line intersecting (*i*_1_, *j*_1_) and (*i*_2_, *j*_2_), the first square root factor is the Euclidean distance between the two points and *ρ* ≥ 1 is a stretching factor for angular coefficients deviating from *π*/4. *d*(⋅) grows as *θ* approaches 3*π*/4 or − *π*/4, i.e. the anti-diagonal orientation. For instance, *d*_*ρ*_((*i*, *j*), (*i*+*k*, *j*+*k*)) = *k* for any *ρ*, *d*_*ρ*_((*i*, *j*), (*i*+*k*, *j* − *k*)) = *ρk* and $d_{\rho }\left(\left(i,j\right),\left(i,j+k\right)\right)=[1+\frac{\sqrt{2}}{2}\left(\rho -1\right)]k$.

**Fig 6:**
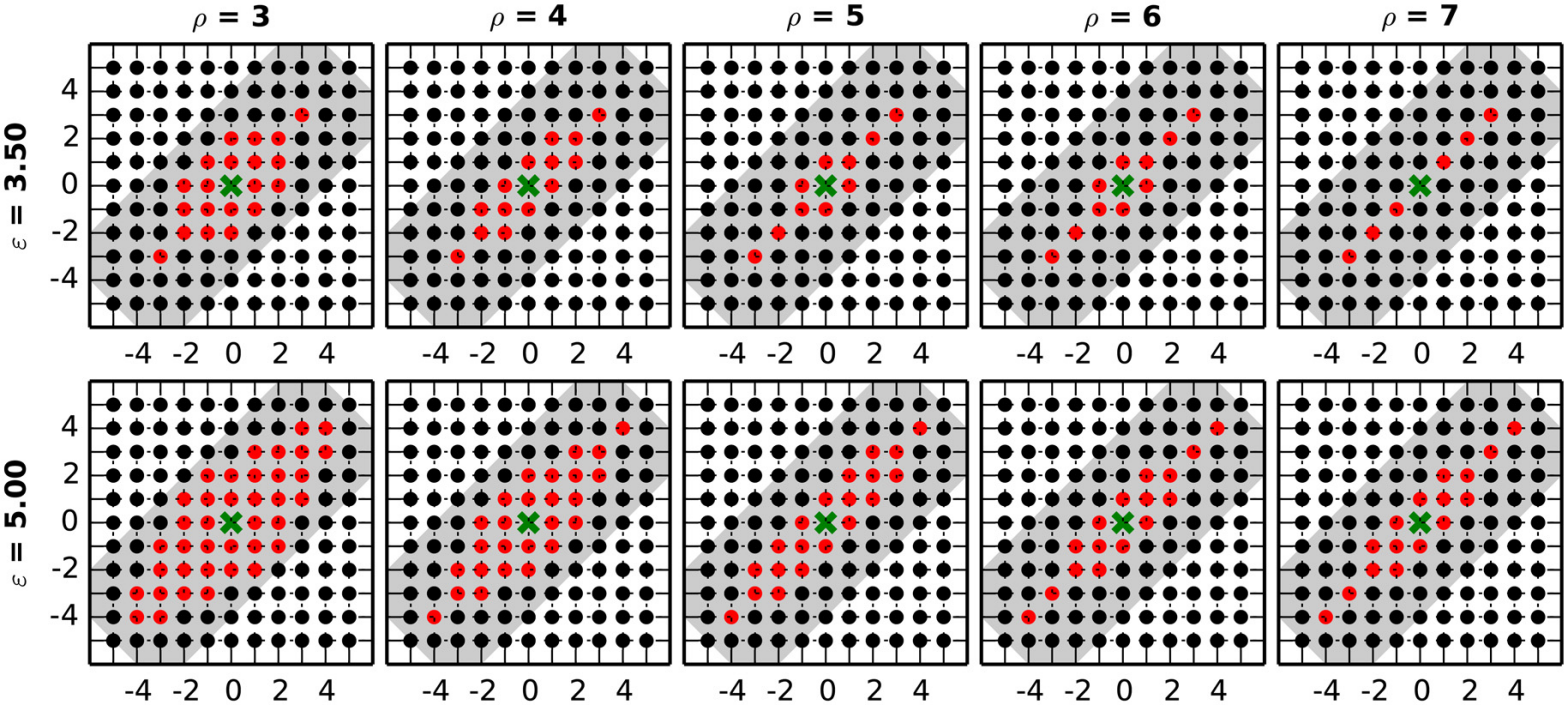
Neighborhood of SSE entries in DBSCAN clustering. Each panel shows, for a different choice of the stretching coefficient *ρ* and of the maximum distance *ε*, the entries falling inside (red dots) and outside (black dots) the ellipse defined by the elliptical distance. The ellipse is centered around the entry *M*_*ij*_ (green cross, here marked at position (0, 0)). Entries inside the ellipse are considered neighbors of *M*_*ij*_ by the DBSCAN clustering algorithm. The gray shaded area represents the kernel, centered around the position of *M*_*ij*_, used to build the joint probability matrix *J*. The ellipse should be contained within the kernel centered around the same position.

We set *ρ* = 5 and, based on the distance *d*_5_(⋅, ⋅) of their positions in the matrix, group all entries *I*_*ij*_ with *M*_*ij*_ = 1 (see Eq 8) into clusters via a density based scanning (DBSCAN) algorithm [14]. The algorithm considers two entries as part of the same neighborhood if their distance is not larger than a maximum value *ε*. Neighborhoods sharing an entry are joined together and eventually classified as a cluster if they contain a minimum number *l*_0_ of entries. We set *ε* = 3.5, thus allowing for a maximum of ⌊*ε*⌋ − 1 = 2 holes between two consecutive entries of a DS along the main diagonal, and *l*_0_ = 3, thus requiring a DS to reflect at least 3 repeated synchronous events. The elliptical neighborhood used when clustering should be contained into the kernel used to build the joint probability matrix *J*. The reason is that the first defines the “immediate” neighbors of the entry, while the second is meant to cover all entries that may belong to the same DS. Thus, the parameter *ε* should not be chosen larger than the kernel length *l*_K_, while the stretching coefficient *ρ* should be chosen such that the shorter axis of the ellipse fits into the kernel width *w*_K_. The values *ε* = 3.5 and *ρ* = 5 we set for the validation satisfy these requirements. Fig 6 illustrates the matrix entries falling inside the ellipse (red dots) for various choices of the parameters *ρ* and *ε*, and the kernel (gray area) centered around the same entry. Entries in the matrix *M* not belonging to any cluster are discarded as events that do not reflect repeated SSE activity.

Fig 3D shows the *cluster matrix* *C* assigning value 1 (colored in black) to entries belonging to a cluster, and 0 (white) to the others. The matrix contains two clusters composed of 5 entries each, corresponding to the 5 synchronous events highlighted in Fig 1A.

### Rate estimation methods

Calculating the entries of the probability matrix as given by Eqs 2 and 3 requires the knowledge of the firing rate of each neuron over time. Firing rate estimation is a problem that has been targeted by a number of studies.

The peri-stimulus time histogram (PSTH, [15]) is an estimate of the firing rate performed by discretizing time into adjacent bins and by counting the number of spikes falling into each bin. The bin width for the PSTH is typically larger (tens of ms) than the one used to define synchrony, as firing rates change on a slower time scale. The larger the bin width, the coarser but less biased the estimate. Normalizing the spike count in each bin by the bin width yields the rate of the process, i.e. the number of spikes per time unit.

Kernel convolution [16] replaces each spike with a kernel (a probability density function) centered around the spike time and estimates the firing rate by the sum of these distributions. Formally, this is done by representing the spike time by a Dirac delta function centered around the spike time t* and by convolving it with the kernel. Following a standard choice, we specify the kernel as a normal distribution with assigned standard deviation *σ*, truncated at ±2.7*σ* to yield a finite support. When setting the kernel width w* = 5.4*σ* to a fixed value, we employ w* = 200 ms.

Both PSTH and kernel convolution can be applied to the case when multiple independent, identically distributed trials of the activity of a neuron are available, by averaging the estimates obtained for each trial. For identical bin and kernel widths, the PSTH typically better represents sharp changes in the firing rate from one bin to the next, while kernel convolution yields smoother curves.

Both estimates are parametric and require the choice of a bin- (kernel-) width. Methods have been recently proposed to determine the optimal bin- or kernel-width by minimizing the error between the true (unknown) rate and its estimate in some statistical sense (see [17, 18]). These methods have been shown to outperform their fixed width variants and are particularly helpful when analyzing parallel spike train data from different neurons with different rates, where the optimal bin width varies across neurons.

In the “*Results*” we compare the performance of ASSET employing either PSTH or kernel convolution or optimized-width kernel convolution [18] estimates of the firing rate profiles over an increasing number of trials.

## Results

We propose here an extension of the method presented in [12] that visualizes repeated temporal sequences of synchronous events (SSE) as diagonal structures (DS, a sequence of large values along a diagonal) within an intersection matrix *I*. *I*_*ij*_ represents the number of neurons which have spikes both in time bin *i* and time bin *j* (see Fig 1 and Eq 1 for the formal definition). We map the intersection matrix *I* into a probability matrix *P* which contains at each position *P*_*ij*_ the probability of an overlap lower than *I*_*ij*_ between the spike trains at the two time bins (Fig 3A). The calculation is derived analytically under the null hypothesis *H*_0_ that the spike trains are Poisson and independent. We then further compute the joint probability matrix *J* whose entries *J*_*ij*_ represent the cumulative joint probability of neighbors of *I*_*ij*_, again under *H*_0_ (Fig 3B). Set two statistical thresholds *α*_1_ < *α*_2_, each entry such that both *P*_*ij*_ > *α*_1_ and *J*_*ij*_ > *α*_2_ hold is classified as a potential member of a DS. Technically, this is done by building a masked matrix *M* such that *M*_*ij*_ = 1 if the two conditions hold, and *M*_*ij*_ = 0 otherwise (Fig 3C). Entries in *M* that take value 1 are finally clustered into individual DSs or discarded as isolated entries based on their reciprocal distance, thus yielding a cluster matrix *C* (Fig 3D).

The positions (*i*, *j*) of entries composing each DS, which can be obtained from the matrix *C*, allow us to reconstruct the synchronous events forming the associated repeated SSE as the intersection of the neurons active at bins *i* and *j*.

We investigate the performance of the proposed method on simulated test data. The data are generated by stochastic simulations of *N* parallel spike trains (here we chose *N* = 100) over a time period of 1*s*. We define 10 types of background spiking activity, differing by the marginal properties of the spike trains (firing rate profile and ISI distribution) and the correlations among the spike trains. We use these data to determine the false positive (FP) rate of the method in cases where SSEs are not included in the data. Then we enrich the data with two occurrences of an SSE composed of *l*_SSE_ = 7 synchronous events, each event involving *ξ*_SSE_ = 5 neurons with IDs 1 − 5, 6 − 10, …, 31 − 35, and investigate therein the true positive (TP) and FP rates. In data containing SSE activity, a found SSE is considered as an FP if it is not composed of (or it is only partly composed of) the events forming the embedded SSE. Details are given below. For the diversity of data types we test the power of the method, further identify critical cases, and suggest solutions.

### Test data

To assess the quality of the method, we first measure its performance in the case when all assumptions entering the derivation of Eq 2 (alternatively Eq 3) and Eq 6 are met. In addition, we test the robustness of the statistics of the method with respect to deviations from these assumptions which are typically found in experimental data. In particular, we investigate how the following features of the data affect the performance of the method and test if these lead to false positive (FP) outcomes. The first four features relate to aspects of firing rates, such as various types of non-stationarities and rate correlations, i.e. correlations between the spike trains on a slower time scale than SSEs. The last two features relate to spike synchrony, however with a different organization than in SSEs.

**Fig 7:**
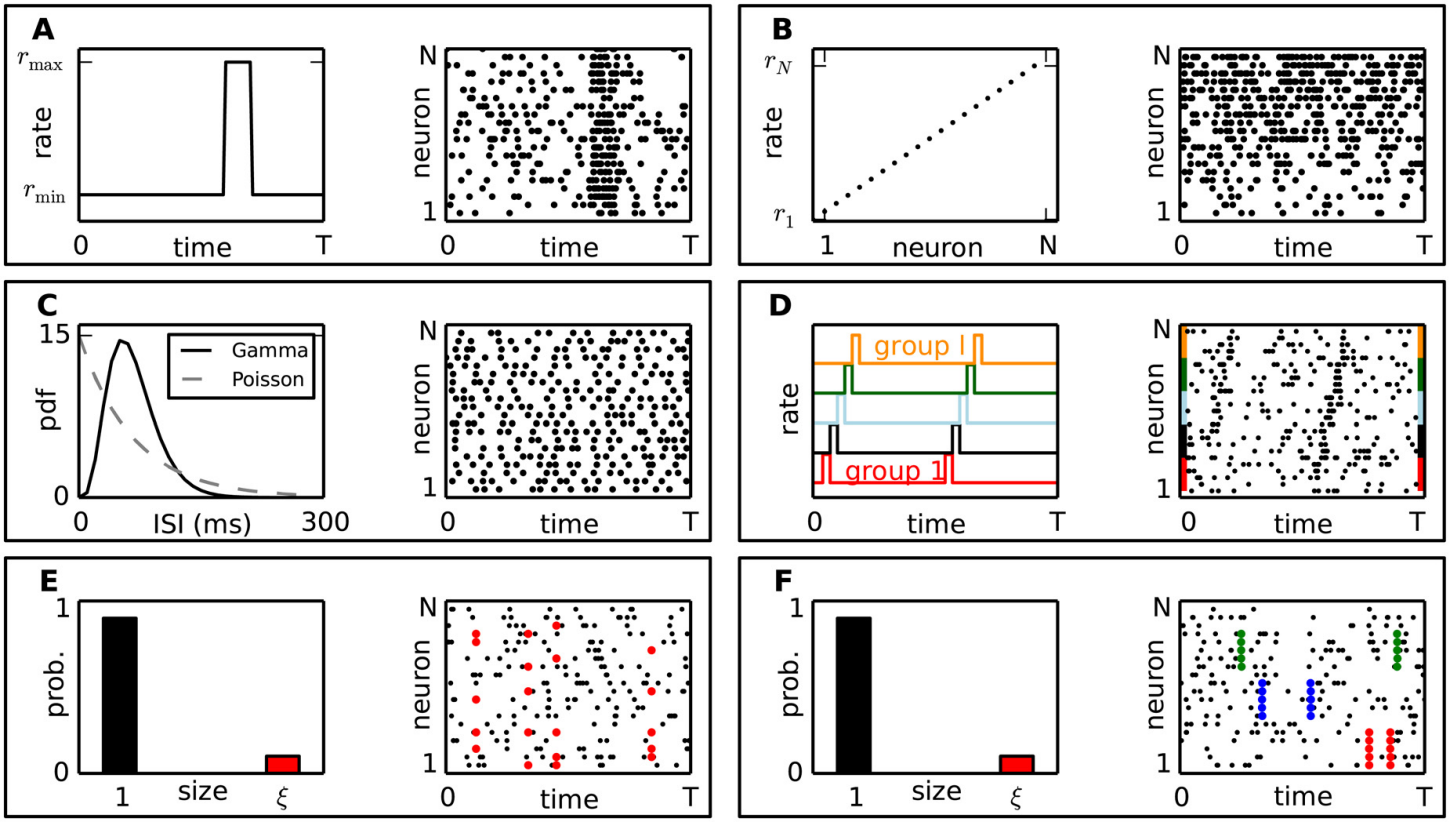
Different features of the data. Each box illustrates one feature (from A to F) of the test data (left column) and an associated exemplifying raster plot (right column). The dots in the raster plots mark the occurrence time of each spike. **(A)** Variability of firing rates over time. The underlying rate profile is shown on the left. **(B)** Heterogeneity of the firing rates across neurons: the different stationary firing rate levels are marked on the left. **(C)** Gamma-distributed ISIs: the underlying Gamma distribution (solid line) is contrasted to a Poisson distribution (dashed). **(D)** Short lasting, sequential rate jumps (different colors) coherent within groups of neurons, repeat identically at two time points. **(E)** Population synchronization: the occurrence probability of the size of the synchronous, but randomly selected spike events, is shown in red, the occurrence probability of independent background spikes (size 1) in black. **(F)** Intra-group synchronization: the complexity distribution is identical to the case of population synchronization in panel E, but the events involve specific groups of neurons (each group is marked in the dot display by one color).

1. Variability of firing rates over time by means of a sudden rate jump that is coherent across all neurons (Fig 7A).
  Firing rate changes over time are the basic observation of experimental data, in particular in response to an external stimulus or in relation to behavior. Neurons reacting to a common stimulus often exhibit a co-modulation of their rates. Ignoring or mis-estimating such rate changes is a typical generator of false positives in synchrony analyses [19–21]. We thus investigate a worst case scenario of coherent and instantaneous rate changes.
2. Heterogeneity of the firing rates across neurons (Fig 7B).
  The firing rates of simultaneously observed neurons typically differ. Although we estimate the firing rates on a neuron by neuron basis, we still want to ensure that the method can cope with this rate variability. Such variability combined with cross-trial variability was shown to be a strong generator of FPs (see e.g. [21]).
3. Increased regularity of the spiking activity compared to the Poisson assumption, by means of Gamma-distributed ISIs (Fig 7C).
  Deviations from Poisson statistics in terms of ISI regularity, which is observed in experimental data [22–24], was shown to bias synchrony analysis methods [20, 25] that, like ASSET, assume Poisson spike trains in the null hypothesis.
4. Short lasting, simultaneous rate jumps in a group of neurons, propagating to other groups in a sequence; this “chain” of rate jumps re-occurs at a later time (Fig 7D).
  This model mimics a rate propagation model, instead of spike synchrony propagation as represented by SSEs. In the ongoing debate on rate coding vs temporal coding [26–28] it was proposed that coherent short-lasting firing rate changes at the input of neurons would be as efficient in bringing neurons to emit a spike as synchronous input. Whether rate correlation and spike synchrony can be distinguished mathematically is being debated (see e.g. [29] vs [30]). Here we ensure that ASSET can distinguish sequences of coherent rate changes from SSE activity when using suitable statistical thresholds.
5. Population synchronization, represented by the occurrence of synchronous spike events at random time points, each involving a random selection of neurons (Fig 7E).
  ASSET operates under the null hypothesis of spike train independence given the estimated firing rate of each neuron. Thus, fine temporal correlations are not incorporated in the null hypothesis. However, studies of recurrent neuronal networks show the presence of weak temporal correlations [31], as those caused for instance by the recurrent connectivity in the network. To study their effect on the performance of our method, we generate test data that contain population correlations with unspecific neuronal compositions. As a correlation model we choose the compound Poisson process, which inserts synchronous spikes at a predefined occurrence rate in randomly selected sets of neurons, rather than in specific groups of neurons as for the SSEs.
6. Intra-groups synchronization, where multiple disjoint groups of neurons exhibit synchronous spiking within the group, at independent points in time for each group (Fig 7F).
  This model represents a second type of correlation structure at fine temporal scale differing from SSE activity. Here, synchronous spike events of specific groups of neurons exist in the same number (7) and size (5) as for the embedded SSE, but in contrast to the SSEs are not emitted in a temporal sequence. This type of correlation was explored already in [32], were it was shown to produce in the intersection matrix isolated high-valued entries, but no DSs. We test here whether this holds true for our more advanced method.

**Fig 8:**
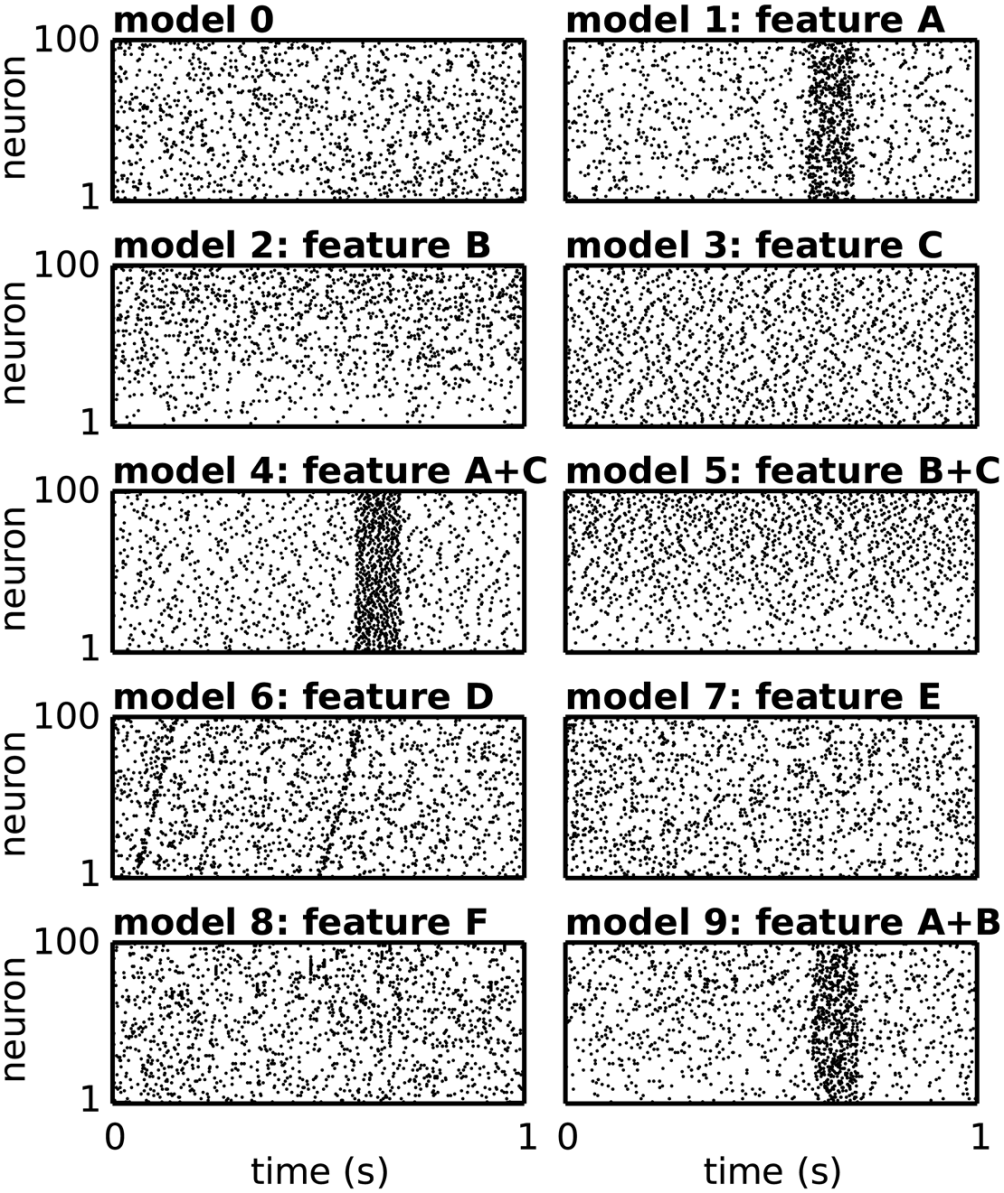
Spiking activity of the stochastic models. Examples of population raster plots of the spiking activity of 100 neurons (vertical axis) over time (horizontal axis), for each stochastic model 0 to 9. Model 0 consists of independent Poisson spike trains with firing rates that are stationary over time and identical across neurons. The other models destroy one or more of these aspects by including some of the features A to F, as listed in Table 1.

For the concrete test cases we formulate 10 different stochastic models of spiking activity (see Fig 8 as examples of the realizations), each including only one or a combination of the above-mentioned features, as summarized in Table 1. We use these models to generate background activity into which we subsequently embed the spiking activity corresponding to a repeated SSE. We provide here the definition of each model.

**Table 1:** Features embedded in the stochastic models. The crosses mark the features (A to F) included and combined in each model (0 to 9) to simulate the background activity of the test data.

	model id									
	0	1	2	3	4	5	6	7	8	9
A: firing rates varying over time		x			x					x
B: heterogeneous rates across neurons			x			x				x
C: high regularity of ISIs				x	x	x				
D: rate propagation							x			
E: population synchronization								x		
F: intra-groups synchronization									x	

#### Model 0—Independent Poisson

*N* = 100 independent Poisson spike trains having identical, stationary firing rates *λ* = 15 Hz.

#### Model 1—Poisson with simultaneous rate jump

*N* = 100 Poisson spike trains having an identical, time-varying firing rate profile λ(t) = 10 Hz + 50 Hz · 1_{600ms<*t*<700ms}_. Thus, all spike trains undergo a simultaneous sudden rate excursion of +50 Hz between times *t*_1_ = 600 ms and *t*_2_ = 700 ms. The average rate over the 1 s simulation period is 15 Hz, as in model 0.

#### Model 2—Heterogeneous, time-stationary Poisson

*N* = 100 independent Poisson spike trains with heterogeneous firing rates across neurons ranging from 5 Hz to 25 Hz. Neuron *k* has stationary firing rate $\lambda_{k}=5+\frac{20}{99}k\;\text{Hz}$, *k* = 0, 1, …, 99. The population-averaged firing rate is 15 Hz, identical to models 0 and 1.

#### Model 3 / 4 / 5—High inter-spike interval regularity

Same as models 0 / 1 / 2 respectively, but the spike trains have marginally Gamma distributed inter-spike intervals (ISIs) with shape factor *α* = 5 (*α* = 1 for the Poisson case) and mean parameter *μ* = 1/15 s

$$\begin{array}{c} P\left(\text{ISI}=\tau \right)={\left(\frac{\alpha }{\mu }\right)}^{\alpha }\frac{\tau^{\alpha -1}e^{-\alpha \tau /\mu }}{\Gamma (\alpha )}. \end{array}$$

The Gamma distribution determines ISIs having mean length *μ* (firing rate: 1/*μ* = 15 Hz), standard deviation $\sigma =\mu /\sqrt{\alpha }$ and thus a coefficient of variation $\sigma /\mu =1/\sqrt{\alpha }$. The larger the *α*, the more regular the ISIs compared to their mean value.

#### Model 6—Fast rate-jump propagation

*N* = 100 independent Poisson spike trains, organized in 20 groups of 5 spike trains each. The first group experiences a sudden, simultaneous rate jump from *λ*_1_ = 14 Hz to *λ*_2_ = 100 Hz at two times *t*_1_ = 50 ms and *t*_2_ = 500 ms, lasting 5 ms (one time bin) each time. Then the second group undergoes the same rate jump 5 ms (one bin) later, and so on. The *l*-th group experiences the rate jump at time *t*_1_ + 5(*l* − 1) ms and *t*_2_ + 5(*l* − 1) ms, *l* = 1, …, 20.

#### Model 7—Population synchronization

*N* = 100 spike trains having all stationary firing rates λ = 15 Hz. From time to time, 5 randomly selected neurons fire synchronous spikes, with a frequency that yields a mean pairwise correlation coefficient among any two spike trains of *ρ* = 0.01. The rest of the spiking activity is mutually independent. Formally, the model is described by a Compound Poisson process (CPP; [33, 34]) with a two-peak amplitude distribution *A*(⋅) such that *A*(1) = 0.938, *A*(5) = 0.062 and *A*(*ξ*) = 0, ∀*ξ* ≠ 1, 5. Each neuron has an average participation rate in synchronous events of 4.63 Hz.

#### Model 8—Intra-group synchronization

Multiple Single-Interaction Process (mSIP, see [32, 35]; cf. [36] for details on the basic SIP model) comprising 65 independent neurons plus 7 groups (SIPs) of 5 neurons each, for a total of *N* = 100 neurons. Each SIP exhibits synchronous activity in two randomly selected time bins, independent for each group. Thus, the resulting data contains 7 disjoint repeated synchronous events of size 5. This is the same as for the case when we inject a repeated SSE into the data, with the difference that the synchronous events do not form a temporal sequence here. Each neuron has a total firing rate λ = 15 Hz, stationary over time.

#### Model 9—Time-varying heterogeneous Poisson

*N* = 100 independent, marginally Poisson spike trains whose firing rates are heterogeneous across neurons and non-stationary over time. Spike train *k* has rate profile

$$\begin{array}{c} \lambda_{k}\left(t\right)=\left(5+\frac{10\cdot k}{99}\right)\text{Hz}+50\;\text{Hz}\cdot 1_{\left\{600\;\text{ms}<t<700\;\text{ms}\right\}}, \end{array}$$

*k* = 0, 1, …, 99. Thus, the spike trains have baseline firing rates ranging from 5 Hz to 15 Hz and experience a coherent rate jump of +50 Hz between 600 ms and 700 ms. The time- and population-averaged firing rate is 15 Hz. This model mixes non-stationarity and heterogeneity of firing rates individually characterizing models 1 and 2, respectively.

Fig 8 shows example raster plots of the activity associated to each of these stochastic models, without additional injection of SSE activity.

### Validation of the method on test data

#### Significance of the two statistical tests

**Fig 9:**
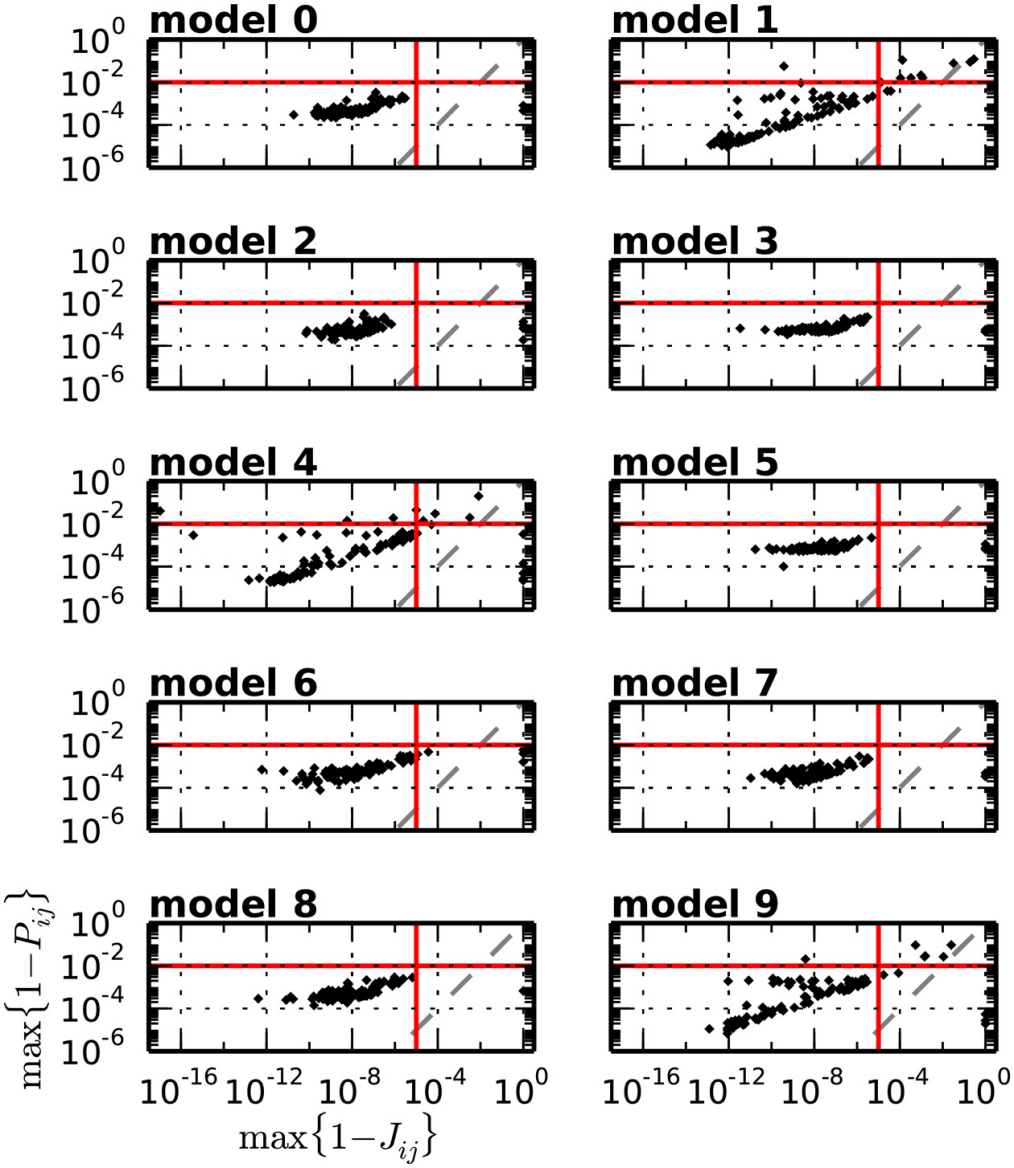
Scatter of the upper-tail probabilities of the two tests in the presence of a repeated SSE. The upper tail probability of the first test (y-axis) in relation to that of the second test (x-axis) for all models 0, …, 9. Each model is repeated 100 times and in each repetition an SSE composed of 7 links and 5 neurons/link is injected at two random points in time. The represented values max(1 − *P*_*i*_*k*_, *j*_*k*__) and max(1 − *J*_*i*_*k*_, *j*_*k*__) are taken each as the maximum (i.e. least significant) significance value across all entries $S_{DS}:=\left\{\left(i_{k},j_{k}\right),\;k=1,2,\ldots ,7\right\}$ of the intersection matrix composing the embedded DS. The figure shows for each model the cloud of the 100 points (max(1 − *J*_*i*_*k*_, *j*_*k*__), max(1 − *P*_*i*_*k*_, *j*_*k*__)), one per simulation. The red lines mark the significance levels 1 − *α*_1_ (horizontal) and 1 − *α*_2_ (vertical) for individual tail probability values 1 − *P*_*ij*_ and tail joint probability values 1 − *J*_*ij*_, respectively.

Eq 3 provides an analytical expression of the cumulative probability *P*_*ij*_ of the intersection value *I*_*ij*_ under the null hypothesis *H*_0_ of independent, marginally Poisson spike trains. The complementary test p-value 1 − *P*_*ij*_ represents the probability that the intersection between bins *b*_*i*_ and *b*_*j*_ is larger than or equal to *I*_*ij*_. Analogously, Eq 6 provides the joint tail probability 1 − *J*_*ij*_ of neighbors of *P*_*ij*_. For positions (*i*, *j*) of the intersection matrix corresponding to a real DS, *J*_*ij*_ should take values which are orders of magnitude lower than *P*_*ij*_, as it represents the joint significance of multiple rather than individual repeated synchronous events. To confirm this, we simulate data from each background activity model 0 to 9 (see Fig 8) for 100 realizations. For each realization, we additionally inject at two points in time, chosen at random, an SSE composed of 7 links and 5 neurons/link, resulting in a real DS composed of a set of 7 entries $S_{DS}:=\left\{\left(i_{k},j_{k}\right),\;k=1,2,\ldots ,7\right\}$ in the intersection matrix. We estimate the firing rate profile of each neuron from single spike trains by kernel convolution (boxcar kernel, kernel width: 200 ms), segment the data in 5 ms bins and calculate the matrices *P* and *J*. Given the large number of neurons involved, we need to rely on Le-Cam’s approximation of the probability mass function of the intersection values (see Eq 3) to compute *P*. We then extract from each matrix the tail probabilities 1 − *P*_*i*_*k*_, *j*_*k*__ and 1 − *J*_*i*_*k*_, *j*_*k*__ of the entries forming the embedded DS, and consider the largest (i.e. least significant) ones, max_*k*_(1 − *P*_*i*_*k*_, *j*_*k*__) and max_*k*_(1 − *J*_*i*_*k*_, *j*_*k*__). Fig 9 shows for each model a cloud of the 100 points (max_*k*_(1 − *J*_*i*_*k*_, *j*_*k*__), max_*k*_(1 − *P*_*i*_*k*_, *j*_*k*__)), one per simulation. As expected, for entries belonging to the true DS the joint significance *J*_*ij*_ is orders of magnitude higher than the individual significance *P*_*ij*_. For this reason it makes sense to set the respective statistical thresholds *α*_2_ and *α*_1_ such that *α*_2_ > *α*_1_. In particular, on the basis of the scatter plots in Fig 9, which show that most tail probabilities 1 − *P*_*i*_*k*_, *j*_*k*__ and 1 − *J*_*i*_*k*_, *j*_*k*__ are lower than 10^−2^ and 10^−5^ respectively, we set *α*_1_ = 0.99 and *α*_2_ = 0.99999.

#### True positive and false positive DSs

For each simulation of models 0 to 9 we identify statistically significant entries (*i*, *j*) in the intersection matrix. We then cluster these entries into diagonal structures using the DBSCAN algorithm, as explained in “*Methods*”. Table 2 summarizes the parameter values used in the analysis.

**Table 2:** Analysis parameters. Parameters of the ASSET method employed for the analysis of stochastic and network data. Each column shows the parameters employed for the corresponding step of the method.

intersection matrix		statistical assessments		DBSCAN clustering	
parameter	value	parameter	value	parameter	value
Δ: bin size	5 ms	*α*_1_: 1^*st*^ threshold	0.99	*ρ*: stretch coeff.	5
*l*: kernel length	5	*α*_2_: 2^*nd*^ threshold	0.99999	*ε*: max distance	3.5
*w*: kernel width	5			*s*: min cluster size	3
*d*: # largest neighbors	5				

**Fig 10:**
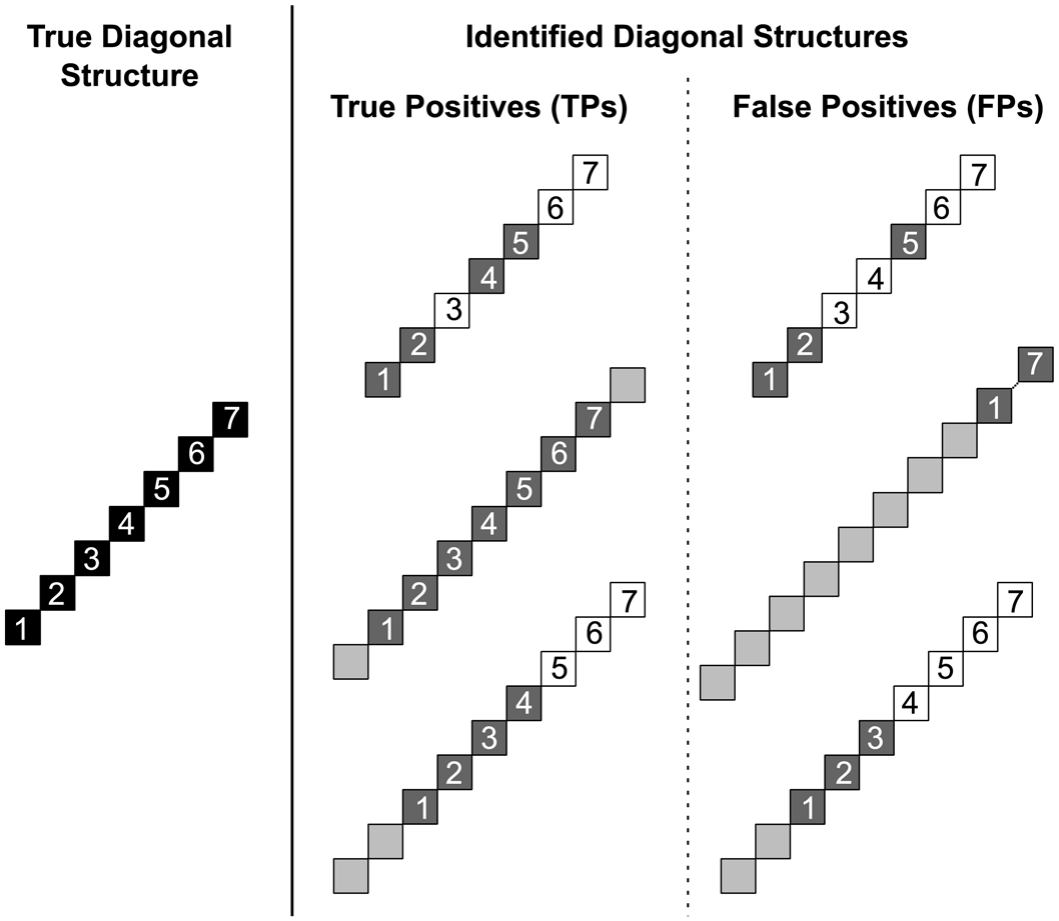
Illustration of true positive and false positive DSs. **Left:** DS composed of 7 entries, resulting from the two occurrences of the SSE embedded in the test data. **Middle, right:** A found DS in the cluster matrix can contain entries that belong to the true DS (dark gray) or not (light gray), and may miss entries of the true DS (white). **Middle column:** Different types of TP DSs found in data: contained in (*top*), containing (*middle*) and partially overlapping with (*bottom*) the true DS. **Right column:** Different types of FP DSs found in data: contained in (*top*), containing (*middle*) and partially overlapping with (*bottom*) the true DS.

We define a DS found in stochastic simulations containing SSE activity as a TP if it contains at least 50% of the entries of the true DS, and if at least 50% of its entries belong to the true DS. If either requirement is not met, or if the data do not contain embedded SSE activity in the first place, the found DS is classified as an FP. Fig 10, left, illustrates the entries composing a true DS in data with embedded SSE activity. Fig 10, right, illustrates FPs arising from the fact that the found DS contains less than 50% of the entries composing the true DS (top, first requirement not met), or contains more than 50% of entries not belonging to the real DS (middle, second requirement not met). The central column in the figure similarly illustrates different types of TPs, when both criteria are satisfied. Note that both TP and FP DSs can be contained in the real DS (top), contain it (middle) or partially overlap with it (bottom).

**Fig 11:**
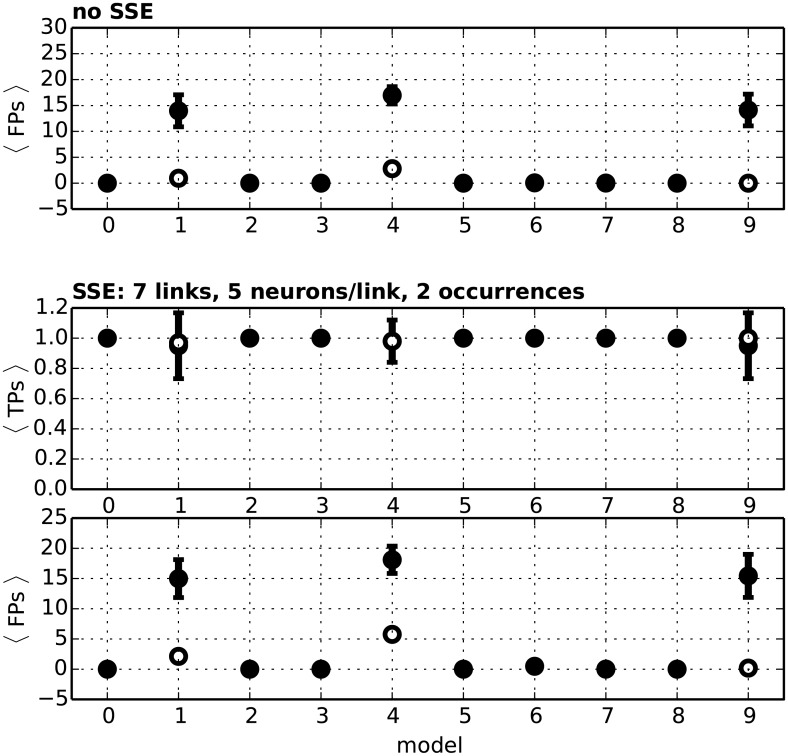
Performance of the method for different models of background activity. The 10 different models of background (SSE-free) activity, numbered from 0 to 9, are simulated 100 times each. **Top panel:** Average number of FPs found in the data when estimating the firing rates of individual spike trains by kernel convolution (filled circles) and when using the theoretical rate profiles that underlie the generation of the test data (empty circles). Bars indicate the standard deviation. **Center and bottom panel:** Average number of TPs and FPs found in the data containing 2 repetitions of an SSE of *l*_SSE_ = 7 synchronous events involving ξ_SSE_ = 5 neurons each.

Fig 11, *top*, shows the FP rate (i.e. average number of FPs over the 100 simulations) in each test data model introduced above, for the case where no SSE is embedded in the data (therefore, each found DS is an FP). The middle and bottom panels show the TP and FP rate for the corresponding cases where two occurrences of an SSE are embedded in the data. Optimal performance is achieved in SSE-free data when the FP rate, which can take values between 0 and +∞, is 0. In data with embedded SSEs the TP rate, ranging between 0 and 1, is optimally 1.

#### Assumptions of null hypothesis met

When the null hypothesis of Poisson and independent spike trains holds, and the firing rates are stationary over time (model 0, identical rates across neurons, and model 2, different rates) the performance is high both in terms of TP rate (= 1.00) and FP rate (= 0.00, both in test data with and without embedded SSEs). Cross-neuron heterogeneity of firing rates does not affect the performance. These results demonstrate the adequacy of kernel convolution, used to estimate the rate profiles on a single spike train basis, when the rates are stationary. The high performance also indicates the efficacy of Le Cam’s approximation (Eq 3) of the true but intractable probability distribution of intersection values (Eq 2).

#### ISI regularity

The optimal TP and FP levels observed in model 0 and 2 are maintained when the spike trains exhibit higher ISI regularity than Poisson (model 3 and 5, respectively). This shows that the method is robust to deviations from the Poisson assumption employed to derive the null distribution.

#### Rate propagation

We further evaluate whether high and short-lasting rate increases affecting a group of neurons simultaneously and propagating from one group to the next in a sequence would be interpreted by the method as the occurrence of an SSE. The main difference between rate propagation and SSE activity is that the first causes neurons to fire stochastically (probability <1) rather than reliably (probability = 1). The first model converges to the second as the rate increase grows to infinity, so that ASSET should find SSE activity in this case. It is otherwise understood as a different model of information coding, which should not yield SSEs.

Using model 6, we simulate an intermediate scenario in which each of 20 groups of neurons successively increases its firing rate from a baseline of 14 Hz to 100 Hz for a period of 5 ms, with an inter-group delay of 5 ms matching the analysis bin width. This fast rate change propagation occurs twice, thus resembling a repeated SSE (see Figs 7D and 8, model 6), but with the firing probability of each neuron of 0.39 during the high-rate regime (instead of 1 for SSE activity and 0.067 during the baseline regime). With these parameters the method yields an FP rate of 0.48 (equivalent to less than 1 FP every 2 model iterations), despite the unrealistically high and sudden rate jump, its coherence across neurons and the fact that the high-rate state perfectly falls into adjacent analysis bins. Deviations from these three features would decrease the false discovery rate substantially. This indicates that the method distinguishes rate propagation from SSE activity as long as the two models are substantially different, but would identify the two when the two models converge.

#### Correlated spike trains

The presence of synchrony involving different groups of neurons each time (model 7) does not harm the performance of the method: the TP and FP rates stay at levels 1.00 and 0.00, respectively. The same results are obtained in data characterized by specific groups of neurons that synchronize their activity within the group, however independently among groups (model 8). These results taken together indicate that, for the range of parameters employed here, the presence of synchronous activity, at the population level or even involving specific groups of neurons, does not affect the performance of the method.

In general, however, one may explicitly include such correlations in the null-hypothesis. To this end we suggest here a Monte-Carlo approach to estimate the probability matrix *J* while taking the observed repeated synchronous events into account. The basic idea is to estimate *J* from surrogates obtained by manipulations of the intersection matrix *I* by destroying the arrangement of rows and columns of *I*. Using this approach, we aim to distinguish synchrony that leads only to isolated high-valued entries in the intersection matrix (non-SSEs) from synchrony that leads to DSs. Concretely, the steps are:

1. Generate *S* (e.g. *S* = 1000) surrogates of the binned discretized data by shuffling the bins randomly.
2. For each surrogate *s*, *s* = 1, 2, …, *S*, compute the corresponding intersection matrix ${\tilde{I}}_{s}$ and derive the associated probability matrix ${\tilde{P}}_{s}$ by Eq 2 or Eq 3, using the original firing rate profiles. This operation preserves all synchronous events in the original data but not their temporal order, and effectively corresponds to an identical random shuffling of rows and columns of *P*. Any DS originally present in *P* is thus destroyed in ${\tilde{P}}_{s}$, thereby implementing the null hypothesis $H_{00}$ that the observed repeated events do not form temporal sequences.
3. Transform each surrogate probability matrix ${\tilde{P}}_{s}$ into a filtered probability matrix ${\tilde{F}}_{s}$ as follows:
  - apply to each entry ${\tilde{P}}_{s;ij}$ a rectangular kernel
  - for each ${\tilde{P}}_{s;ij}$ extract its *d* largest (most significant) neighbors ${\tilde{P}}_{s;ij}^{\left(k\right)}$, *k* = 1, 2, …, *d* and set ${\tilde{F}}_{s;ij}:=1-\prod_{k}(1-{\tilde{P}}_{s;ij}^{\left(k\right)})$.
4. In the same way transform the original probability matrix *P* into a filtered matrix *F*.
5. Compute the significance of each entry *F*_*ij*_ in *F* by comparison with the sample $\left\{{\tilde{F}}_{s;ij},\;s=1,2,\ldots ,S\right\}$, i.e. set
  $$\begin{array}{c} {\tilde{J}}_{ij}=\frac{1}{S}\left|\left\{s:\;{\tilde{F}}_{s;ij}\leq F_{ij}\right\}\right|. \end{array}$$

The matrix $\tilde{J}$ is a Monte-Carlo estimate of the joint probability matrix under $H_{00}$. Its entries ${\tilde{J}}_{ij}$ are statistically significant if ${\tilde{J}}_{ij}>\alpha_{2}$. In summary, if a repeated SSE was present in the data, the surrogate destroys the corresponding DS in *I* and thereby leads to higher (more significant) values in $\tilde{J}$ than if there was no such arrangement in the first place, as e.g. for non-SSE synchrony.

**Fig 12:**
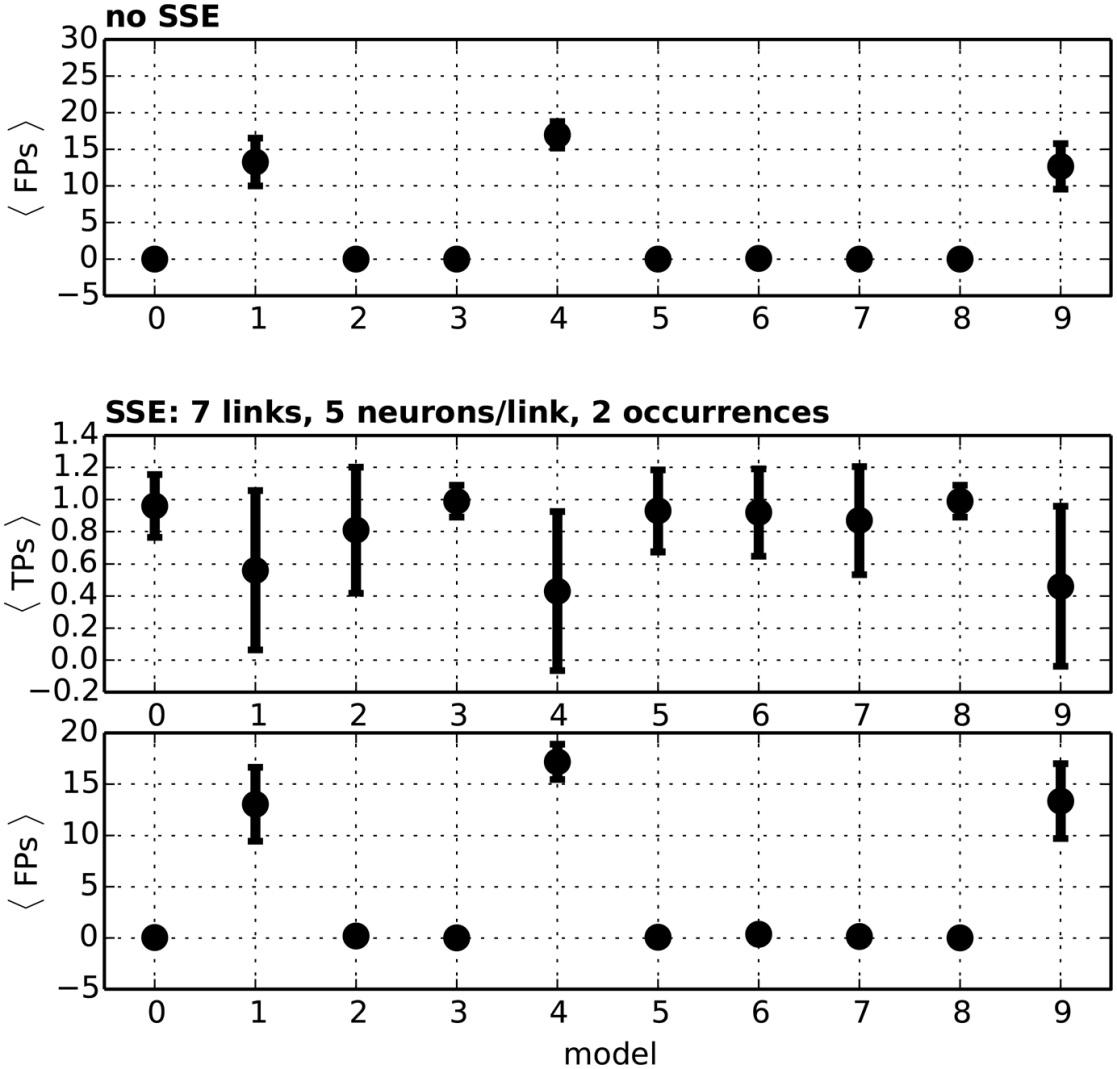
Performance of the Monte-Carlo approach. **Top panel:** Average number of FPs (vertical axis) found over 100 simulations of each model from 0 to 9 (horizontal axis) using the Monte-Carlo estimate $\tilde{J}$ of the joint probability matrix under $H_{00}$. **Middle panel:** Average number of TPs found for each model from 0 to 9 after injecting two repetitions of an SSE (equivalent to one DS in the intersection matrix) with 7 events and 5 neurons/event. **Bottom panel:** Average number of FPs for the same data as in the middle panel.

Fig 12 shows the performance on models 0 to 9 using this Monte-Carlo estimate (and thus working under $H_{00}$). The FP rate is comparable to that obtained using the analytical approach under *H*_0_. Importantly, this holds also for models 6 − 8 where correlations are present, demonstrating that the correlations embedded in the data do not bias the analytical estimates. The Monte Carlo approach shows also decreased TP rate, especially for the cases where firing rates are non stationary (models 1, 4 and 9, TP rate <0.6). The reason is that time bin shuffling effectively destroys the rate profiles and thus ignores rate changes when building the null distribution. Nevertheless, scenarios may be thought of where correlations need to be taken into account by means of the Monte-Carlo approach illustrated above.

#### Time-varying firing rates

We further investigate how time-varying firing rates affect the performance of the method. Model 1 contains independent Poisson spike trains, as in model 0, with the difference that the firing rate profiles exhibit a coherent rate jump from 10 Hz to 60 Hz at time *t*_1_ = 600 ms and back to 10 Hz at time *t*_2_ = 700 ms (see Figs 7A and 8). Similarly, models 4 and 9 combine this rate non-stationarity with Gamma-distributed ISIs (Fig 7C) and with cross-neuron rate heterogeneity (Fig 7B), respectively. In all cases the FP rate increases beyond 14.0 (*filled circles* in Fig 11). The problem resides in the inability to estimate the instantly varying firing rates by means of a fixed-width kernel convolution, which smooths the rate jump over a larger window. Indeed, when using the true rate profiles to compute the statistics, the FP rate drops back to values lower than 2.0 for models 1 and 9, and as low as 5.7 for model 4 which features highly regular ISIs (*empty circles*).

**Fig 13:**
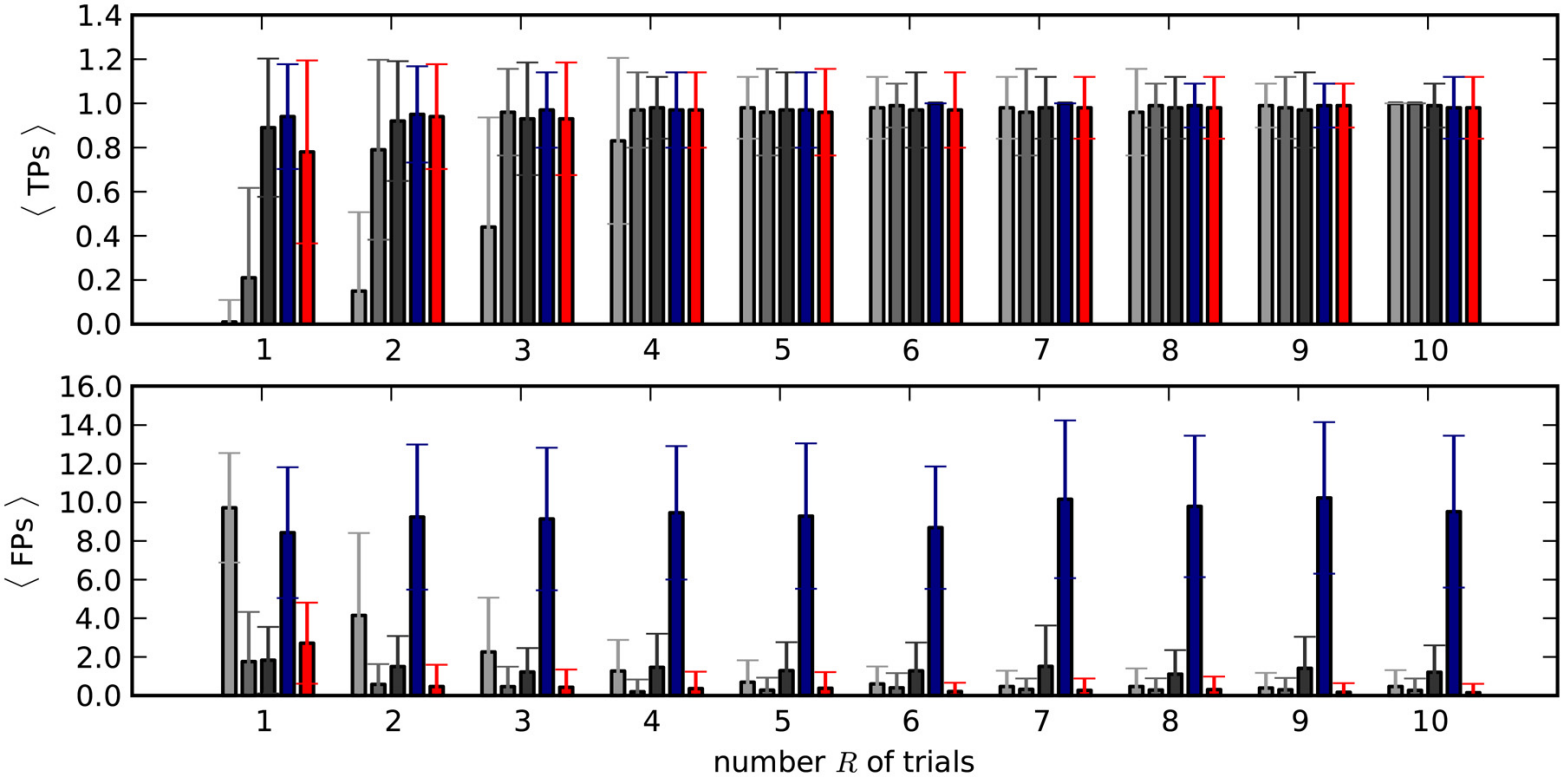
Rate profiles estimated via 3 different methods computed over an increasing number of trials. Average number of TPs (*top*) and FPs (*bottom*) resulting from different firing rate estimation methods, as a function of the number of trials used to estimate the firing rates of individual neurons (horizontal axis). The data are defined by model 4 and enriched with two repetitions of an SSE with *l*_SSE_ = 7 links and ξ_SSE_ = 5 neurons/link. *Gray bars*: PSTH (dark: 20 ms bin width, medium-dark: 10 ms, light: 5 ms). *Blue bars*: kernel convolution with fixed kernel width (200 ms). *Red bars*: optimized-bandwidth kernel convolution.

In realistic scenarios of analysis of experimental data firing rate profiles are not known. However multiple trials, i.e. repetitions of the same stimulus or behavioral condition related to a given trigger event, are often available that make the estimation of the firing rate possible and more reliable. Common tools for firing rate estimation across trials are the peri-stimulus trial histogram (PSTH, [15]) or trial-averaged kernel estimates. We use model 4, which represents a worst case scenario (sudden co-modulation of rates, non-Poisson spiking), to investigate whether cross-trial estimation of the firing rates is accurate enough for our needs since it comprises a worst case scenario (coherent rate jump, non-Poisson). To this end we estimate the firing rate profile of each neuron by simulating its activity *R* times (“trials”), *R* = 1,2,…,10 and computing its time-resolved average rate over these trials using three different estimation techniques (for details, see “*Rate estimation methods*”): PSTH with a bin width of 5, 10 or 20 ms, kernel convolution [16] with fixed kernel width (200 ms) and kernel convolution with an optimized kernel width [18]. Fig 13 illustrates the resulting performance for each method as a function of the number *R* of trials considered for the estimation. The PSTH (gray bars) performs well for a properly predetermined bin width (here the best being 10 ms, resulting in a TP rate >0.9 and an FP rate <0.2 when 3 or more trials are available). The same problem affects kernel convolution with a fixed kernel width, which is here chosen too large, resulting in large numbers of FPs. Instead, the optimized-width kernel convolution solves the problem by determining the optimal kernel width in a statistical sense, and yields here a TP rate >0.9 and an FP rate <0.2 as soon as 3 or more trials are considered. Thus, this proves to be a suitable method for the rate estimation for experimental data, which are typically characterized by strong and fast rate changes, provided that multiple trials are available.

#### Number of neurons and size of the DS

We finally investigate how the power of the method is affected by a reduced number of neurons involved in SSE activity in the total data set. To this end, we repeatedly simulate background activity as in model 0 varying the total number *N* of neurons from 50 to 500, and add two occurrences of an SSE varying the number *l*_SSE_ of links (from 3 to 7) and the number *ξ*_SSE_ of synchronous spikes per link (from 2 to 5). For each value of *N*, *l*_SSE_ and *ξ*_SSE_ we generate the data 100 times and analyze each of them with ASSET employing the parameters reported in Table 2. Note that the kernel length employed (*l*_K_ = 5) is suboptimal for values of *l*_SSE_ higher than 3, as in those cases the kernel centered at the ends of the DS does not cover the full DS and a longer kernel would yield higher performance.

**Fig 14:**
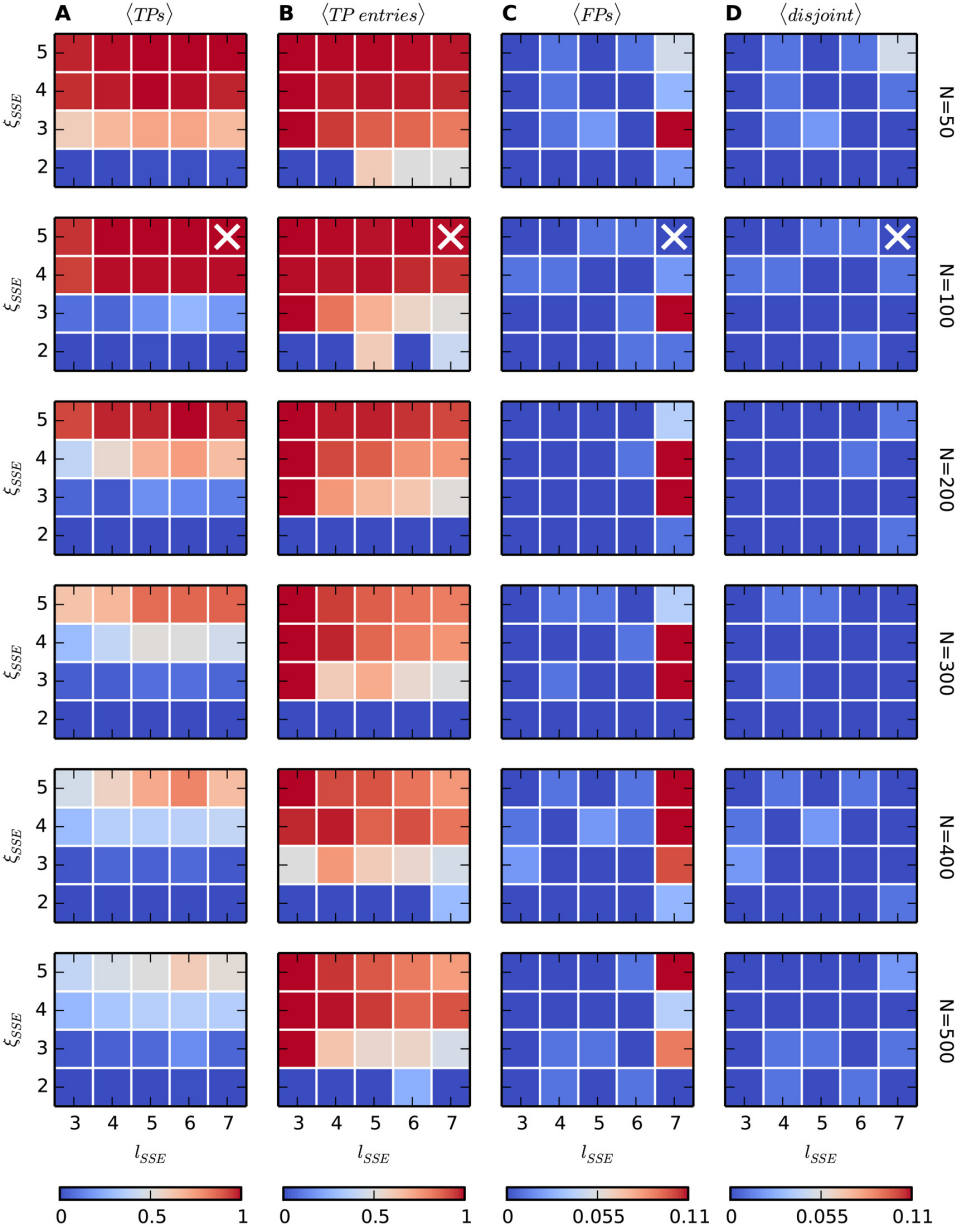
*Performance as a function of l*_SSE_, *ξ*_SSE_ *and N*. **(A)** Average number of TP SSEs (over 100 model simulations) found in data where an SSE with varying number of links (*horizontal axis*, from 3 to 7) and neurons per link (*vertical axis*, from 2 to 5) is injected twice in the activity of *N* otherwise independent spike trains (model 0). *N* varies from 50 to 500 (top to bottom panels). For *N* = 100 the cross at position (*l*_SSE_ = 7, *ξ*_SSE_ = 5) marks the parameters used in the previous calibrations. **(B)** Average number of events of the embedded SSE contained in each found SSE (“true positive events”). **(C)** Average number of FP SSEs (note the different scale of the color map). A FP can either be completely disjoint from the true embedded SSE, or contain part (or all) of its member events (see definition above and Fig 10). **(D)** Average number of found SSEs completely disjoint from the embedded one.

The results are shown in Fig 14. The TP rate (column A) increases for a larger size *ξ*_SSE_ of the synchronous events and a smaller total number *N* of spike trains, as a larger fraction of the total neurons are involved in the SSE. However, the TP rate does not increase substantially with the number *l*_SSE_ of synchronous events composing the embedded SSE, because the kernel has fixed length and does not cover the additional events, thus resulting in stationary joint significance values *J*_*ij*_ of the second statistical test. For low *l*_SSE_, low *ξ*_SSE_ or large *N* the TP rate drops considerably to values lower than 0.5. This is partly due to our strict definition of a TP, which does not include found SSEs containing less than 50% of the synchronous events composing the embedded SSE (true positive events). Fig 14, column B, shows the average fraction of true positive events composing each found SSE. The FP rate (column C) is close to 0 (and always lower than 0.1; note the different scale of the color map) for all investigated values of *l*_SSE_, *ξ* and *N*. Importantly, it does not increase with *N*, which is a relevant feature of the method for applications to large-scale data. For *l*_SSE_ = 7 and *ξ*_SSE_ ≥ 3 we observe occasionally jumps in the FP rate. Almost all of these FPs consist of SSEs which partially overlap with the embedded one, but not enough to be classified as TPs. As a comparison, Fig 14, column D, shows the rate of FP SSEs that are completely disjoint from the true one. This rate is indeed close to 0 for all parameter choices, showing that the increased FP rates shown in column C result from partially true discoveries.

We then investigate the special case of SSEs composed of 1 spike per group only (*ξ*_SSE_ = 1), to test if our method is able to detect spatio-temporal patterns involving no spike synchronization. We inject as before two repetitions of a spatio-temporal pattern, now composed of *l*_SSE_ = 7 spikes with a time delay of 5 ms, into independent data (model 0, composed of *N* = 10 neurons). Because the previous analysis indicates low performance already for *ξ*_SSE_ = 2 for the parameters employed so far, we increase the kernel length to *l*_K_ = 7.

**Fig 15:**
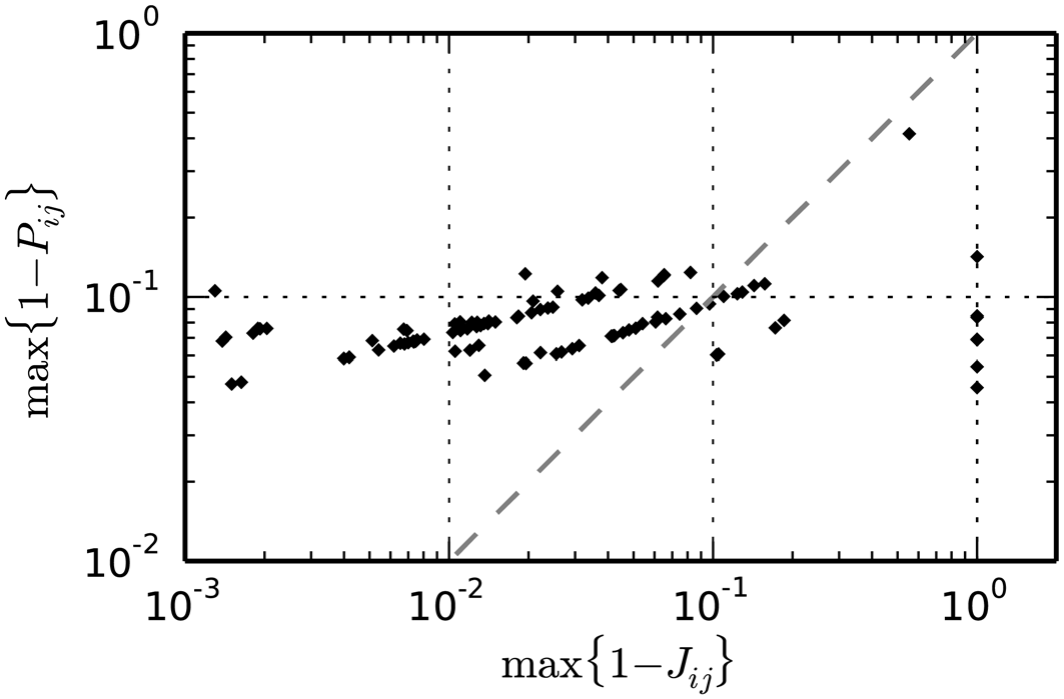
Scatter plot of the upper-tail probabilities of the two tests in the presence of a repeated spatio-temporal pattern. The upper tail probability of the first test (vertical axis) over the upper-tail probability of the second test (horizontal axis) for a reduced version of model 0. The model consists of parallel activity from *N* = 10 independent Poisson spike trains, plus two repetitions of a temporal sequence of *l*_SSE_ = 7 spikes from 7 different neurons (*ξ*_SSE_ = 1). The repeated spatio-temporal pattern generates a DS in the intersection matrix composed of 7 entries $S_{DS}:=\left\{\left(i_{k},j_{k}\right),\;k=1,2,\ldots ,7\right\}$, and corresponding values *P*_*i*_*k*_, *j*_*k*__ and *J*_*i*_*k*_, *j*_*k*__ in the probability and joint probability matrices, respectively. The data are generated 100 times. For each data set and corresponding DS in the intersection matrix, the figure shows a point (max_*k*_(1 − *J*_*i*_*k*_, *j*_*k*__), max_*k*_(1 − *P*_*i*_*k*_, *j*_*k*__)), corresponding to the least significant entries in the matrices *J* and *P* among those composing the DS. The figure shows the cloud of the 100 such points, one per simulation.

As shown in Fig 15, the tail probability of individual entries in the intersection matrix corresponding to the real DS and the joint tail probability of their *d* = 5 largest neighbors are both too large (weakly significant) to yield sufficient test power. A larger number of repetitions of the spatio-temporal pattern may increase the statistical significance to acceptable levels, as commented in “Discussion”. Thus, we conclude that ASSET is not suitable for the detection of spatio-temporal patterns involving no spike synchrony.

### Analysis of a synfire chain network simulation

#### Synfire chain data

Synthetic data from simulations of a balanced neuronal network with embedded overlapping synfire chains (SFCs, [8]) were produced in [12]. The full network comprises 40,000 excitatory and 10,000 inhibitory neurons, with biologically realistic connectivity. 50 synfire chains are embedded, each obtained by selecting 2000 neurons of the network and randomly organizing them in 20 successive groups of 100 neurons each. All neurons in one group are connected to all neurons in the next group in a feed-forward fashion, resulting in high synaptic convergence and divergence. Individual neurons participate in up to 3 SFCs, which therefore overlap. The SFCs are stimulated by injection of a current pulse in the first link of the chain, leading to synchronous spiking activity in the first group of the chain which propagates downstream. The propagation delay between one group and the next is ∼2 ms. The propagation is robust to the presence of noise and does not require all neurons in a link to be active for propagation to the next link [10].

**Fig 16:**
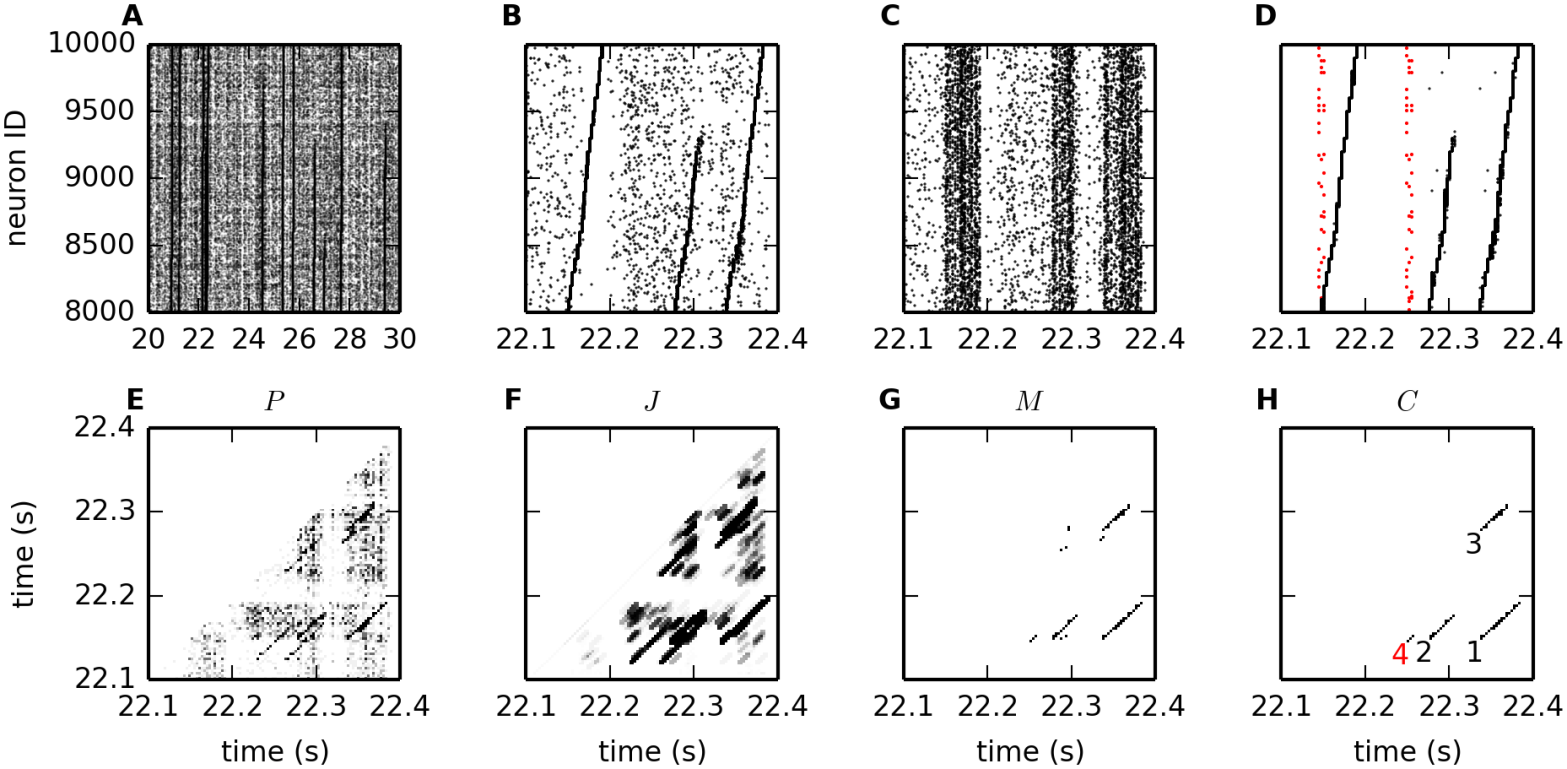
Synfire chain network data and analysis results. **(A-D)** Activity of 2000 neurons (numbered 8001 to 10,000) forming the entirety of an active SFC in a balanced random network [12] composed of 20 groups. Neurons 8001 − 8100 form the first group of the chain, neurons 8101 − 8200 the second group and so on. A subset of the neurons also participate in 3 successive groups of a second SFC in the network. **(A)** Activity over a time window of 10 s (replicated from [12], Fig 2A). **(B)** Enlargement of (A) showing 300 ms of data (replicated from [12], Fig 2B). The SFC under consideration is activated three times in this time interval. **(C)** Same as in panel (A), but with random sorting of the neuron IDs on the vertical axis. **(D)** SSE activity detected by ASSET. In the time interval considered, the two SFCs are stimulated three times (black dots) and two times (red dots), respectively. **(E-H)** Probability matrix *P*, joint probability matrix *J*, masked matrix *M* and cluster matrix *C*. Numbers 1 to 3 and 4 in the cluster matrix mark the significant DSs found by ASSET and associated to the activations of the first and second SFCs, respectively.

We consider the activity of 2000 neurons of the network, having identities 8001 to 10,000, over a time segment of 300 ms. The considered neurons compose the entirety of one of the 50 SFCs. In particular, neurons 8001 to 8100 compose the first link, neurons 8101 to 8200 the second link and so on until neurons 9901 to 10,000, which compose the last link of the chain. Fig 16 shows the activity of these neurons over a time stretch of 10 s (A) and of 300 ms (B,C). Panels A and B are adapted from Figure 2 in [12]. As apparent from panel B, in the considered time window the selected synfire chain is active three times. The first and last time the activity runs through all the 20 links, the second time it propagates only until the 13-th link. Panel C reproduces the same data as in panel B, but with a random shuffling of the neuron identities along the vertical axis. Here, the SFC activity misleadingly appears as a co-modulation of firing rates across neurons.

In addition, some of these neurons also belong to 3 successive groups of a second SFC. The 3 groups comprise 14, 12 and 14 neurons of the population analyzed, respectively. In the considered time period, this second SFC is active twice. Contrarily to the first SFC, the activity of the second SFC does not become apparent in the raster plot of Fig 16B, because the sorting of neuron identities along the vertical axis of the plot does not place neurons belonging to the same group close-by. Neither does this activity reflect in the plot as population co-modulation, because it involves a relatively small fraction (2%) of the population.

#### Analysis of synfire chain data

We analyze the stretch of data illustrated in Fig 16B with ASSET to demonstrate that the method is able to discover repeated synfire chain activation. The activation of each SFC generates an SSE, and each pair of SSEs corresponds to a DS in the intersection matrix. Therefore, the triple activation of the first SFC generates 3 diagonal structures that the method should find in the cluster matrix: one (DS 1, composed of 20 links) is given by the overlap of the two complete runs of the SFC (which involve 20 synchronous events each), and the other two (DSs 2 and 3) are composed of 13 links each, resulting from the comparison of each complete activation with the one stopping at the 13-th group. The double activation of the second SFC, composed of 3 successive synchronous events each, should analogously yield 1 significant DS (DS 4) of length 3. Indeed, the method finds all of these DS, as shown in Fig 16H. The raster plot in Fig 16D shows the SSEs generated by the activation of the two SFCs (black and red dots, respectively; spikes not belonging to the SSEs not shown), which ASSET reconstructs in their entirety.

Table 3, first row, reports for each DS the true number of composing entries and the true distance (in number of bins) between the first and the last event for the first SSE occurrence (*dx*) and for the second SSE occurrence (*dy*). The second row shows the corresponding values found by the analysis. Due to a larger bin width (3 ms) than the inter-link propagation delay (∼2 ms), the found SSEs are shorter than the number of links in the chain: some successive events fall in the same time bin and are therefore merged into a single event. Nevertheless the composing spikes are correctly retrieved, as well as the associated units. Rows 3 to 5 in the table show the median values of matrix entries corresponding to the DS in the intersection matrix, the probability matrix and the joint probability matrix, respectively. As a comparison, the median values of the full matrices were 0, 1 and 1 respectively, indicating the high statistical significance of the entries forming the found DSs compared to the other entries in the matrices. Note that the kernel length employed (length *l*_K_ = 5) is shorter than the number of entries composing the DSs associated to the first SFC (≥9). Nevertheless, these DSs are successfully retrieved.

**Table 3:** Diagonal structures of active synfire chains. The top row shows in bold the true number of repeating synchronous events forming the four pairs of repeating SSEs present in the data. The second row shows the number of entries composing each of the four associated DSs as found by the analysis, and in brackets the distance (in number of bins) between the first and the last event of the first SSE occurrence (*dx*) and the second SSE occurrence (*dy*). The other rows in the table show the median value of the entries in the intersection matrix (*second row*), in the probability matrix (*third row*) and in the joint probability matrix (*fourth row*) corresponding to each DS.

	DS 1	DS 2	DS 3	DS 4
true nr. entries (*dx*/*dy*)	20	13	13	3
nr. entries (*dx*/*dy*)	23 (14/15)	13 (9/9)	18 (10/10)	3 (2/2)
median i-mat value	93	88	53	14
median p-mat value	< 10^−22^	< 10^−22^	< 10^−22^	∼ 6.6 ⋅ 10^−8^
median j-mat value	< 10^−22^	< 10^−22^	< 10^−22^	∼ 3.9 ⋅ 10^−14^

These results taken together demonstrate the ability of the method to retrieve repeated SSE activity, here generated by repeatedly active SFCs, in massively (2000) parallel spike trains. The method yields high significance values even when employing sub-optimal parameters, such as a larger bin size than the inter-link transmission delay or significantly smaller kernel length than the DS length.

### Computational performance

The computational cost of the algorithm is almost entirely determined by the time required to evaluate the joint probabilities defined in Eq 6. The expression involves a nested sum of several terms. The number of terms grows with the number *n* of matrix entries covered by the kernel (determined by the kernel length *l*_*K*_ and the kernel width *w*_*K*_) and with the number *d* of largest neighbors among which the joint significance is computed. These are free parameters of the analysis, whereas features of the data, such as the number of neurons or their firing rates, do not influence this step of the computation. As an estimate, for the values we employed in the manuscript (*l*_*K*_ = 5, *w*_*K*_ = 5, *d* = 5) the evaluation of a single entry took about 10 ms on a single core of a dual AMD 12-core Opteron 6174 machine with 64GB RAM using the Python code provided with this manuscript. Thus, the evaluation of a full matrix *J* of 200 × 200 entries took on average less than 7 minutes. However, the fact that single entries are evaluated independently may be easily exploited by parallelizing the analysis on multi-core machines or computer clusters, where each worker process is assigned to perform the computation for a subset of the matrix entries (see [37]).

## Discussion

Temporal sequences of synchronous spike events (SSEs) have been postulated as a working mechanism of activity propagation in the cortex [38–40]. The present manuscript introduces a novel statistical method for the detection of SSEs in massively parallel spike train data, named ASSET (Analysis of Synchronous Spike EvenTs). The method is inspired by a visual technique first proposed in [12], which represents the repeated occurrence of an SSE as a sequence of large entries along the diagonals of an intersection matrix (diagonal structure, or DS) that indicates for any two time bins the number of neurons firing in both bins. ASSET automatizes the detection process of the original visual technique, assesses the statistical significance of SSEs by exploiting the multiple evidence of its events to derive their joint significance, and determines the structure and neuronal composition of the identified SSEs. In evaluating the null distribution, the method accounts for the temporal profiles of the firing rates of the observed neurons. As such, it detects SSEs which cannot be explained on the basis of rate coding mechanisms, and thus arise from spike correlations on a shorter temporal scale. Rate correlation is understood as a conceptually different mechanism of information processing than spike synchrony (as for SSEs), because it corresponds to stochastic (probability <1) rather than reliable (probability 1) neuron activation [29, 41–43].

We assessed the performance of ASSET in terms of false positive (FP) DSs found in various types of stochastic models which mimicked typical features of neuronal spike trains, such as variable firing rates in time or across neurons, different inter-spike interval distributions, and correlation structures differing from SSEs. We then additionally injected repeated SSE activity in the data to assess the power of the method in terms of true positive (TP) detections. The analysis performs two statistical tests on each pair of time bins, which amounts to tens of thousands of tests for a stochastic simulation of 1 s binned at 5 ms. In addition, entries passing the two tests have to lie close to each other in the matrix in order to be clustered into a common DS. To avoid FPs, the statistical threshold needs to be set to low values (here, 10^−5^). This is possible without incurring into large levels of FNs because the joint tail probabilities associated to the second test are very low (typically <10^−5^, and as low as 10^−12^) for the entries corresponding to the embedded SSEs. Indeed, the method shows high performance, i.e. FP and FN rates both close to 0. We did not need to further correct the statistical thresholds by the amount of tests performed (e.g. by Bonferroni or FDR correction), because already the set of combined requirements that need to be fulfilled to identify a DS (success of the two tests for matrix entries, and proximity of these entries in order to be clustered into a DS) makes the analysis very conservative.

The underlying null hypothesis assumes independent Poisson spike trains, which enables an analytical formulation of the test statistics. The method proves to be robust to deviations from Poissonianity, such as a higher regularity of the inter-spike intervals, which may be observed in experimental data [22, 44]. It is also selectively sensitive to SSEs, but not to other models of spike correlation, such as synchronous events not organized in a temporal sequence. Nevertheless, anticipating scenarios where strong correlations not forming an SSE might indeed bias the statistics, we proposed a Monte-Carlo approach to account for these correlations. To this end, we constructed the null hypothesis by estimating the probability to find a certain degree of pattern overlap from the repeated generation of surrogate intersection matrices. In our test data the Monte-Carlo approach yielded results comparable to the analytical approach, yet at a considerably higher computational cost.

Furthermore, the method was able to distinguish SSEs from repeated precise temporal sequences of sharp, local rate transients from one group of neurons to another (rate propagation). Rate peaks increased the probability of the involved neurons to spike, which remained nevertheless a stochastic event, in contrast to SSE activity. Propagation of rate transients thus generated spike patterns that were different in composition (due to the stochastic activation of neurons) and less precisely timed than SSEs, but more closely resembled the latter as the rate modulation became higher and faster. It is likely that, for extremely high firing rates and very short rate jumps, ASSET would not distinguish the two models. The distinction between rate correlation and synchrony correlation (see e.g. [29]) has been formally questioned in [30]. Users of ASSET may want to identify waves of co-modulating rates like those defined in our model 6 as SSEs, rather than rejecting them. This is possible by decreasing the statistical thresholds *α*_1_ and *α*_2_ to less strict values than those used in this manuscript.

ASSET critically relies on the estimation of the firing rate profile of each neuron to compute the expected overlap of neuron IDs in the intersection matrix and thus to estimate the significance of the observed overlap. Estimating firing rate profiles on the basis of single trial spike data typically requires some kind of sliding window approach, such as convolution of each spike with a temporal kernel [16]. Temporal averaging smears out peaks of the underlying original firing rate that occur on a shorter time scale than the window width, and creates artificial peaks if the window width is excessively short. Single-trial rate estimates obtained by kernel convolution in the presence of time-stationary firing rates yielded high performance of ASSET, but led to impaired performance when the rates were non-stationary in time on a fast time scale, in particular if the rate excursion was coherent across neurons. The reason is that smoothing by convolution underestimates positive rate peaks and thus the expected overlap, yielding FPs. Estimating the firing rates on the basis of trial averages solved the problem. We here tested three such approaches, namely the peri-stimulus time histogram (PSTH, [15]), a trial-averaged kernel convolution with fixed kernel width [16] and a trial-averaged kernel convolution with an optimized kernel width [18]. Already a small number of trials reduced the FP rate considerably for all three methods, although best performance was achieved for the optimized-width kernel convolution. Importantly, cross-trial rate estimation works under the assumption of identical rate profiles across trials. Deviations from this assumption lead to a wrong estimation of the rate in single trials, that is required to calculate the probability matrix, and thereby enhances the FPs. This bias is amplified if neurons exhibit cross-trial variability in a coherent manner [19]. Latency variability is a special instance of cross-trial non-stationarity which causes a mis-estimation around the rate onset. In some cases the onset variability of rates can be corrected for by choosing a more proper alignment of trials, e.g. to the stimulus or behavioral event related to the rate change [45]. If this is not possible, we suggest to generate the probability matrix *P* on the basis of surrogate spike data, e.g. by spike train shifting or spike dithering [20]. The details of such an approach, however, still need to be explored.

We further investigated how the performance of ASSET relates to various other parameters of the SSEs, such as the number of its sequential synchronous events composing the SSE, the number of neurons in each synchronous event, and the total number of observed neurons. SSEs were statistically more significant and therefore easier to detect when they involved a larger fraction of the total neurons. However, they could be retrieved even when employing sub-optimal parameters such as a kernel length smaller than the length of the DS, given that the SSE involved enough neurons. In contrast, the method did not detect spatio-temporal patterns, i.e. a special case of SSE where each event is composed by a single spike only. Spatio-temporal patterns in a more general sense (with different time intervals between spikes) were suggested as signatures of synfire chain activity in data of low numbers of simultaneously recorded neurons [40, 46, 47].

A less constrained model of cortical processing (synfire braid) was proposed in [48], which incorporates synchronous input to individual neurons as in the synfire chain model, however transferred by connections of different temporal delays compensated by differences in their activation times. This model was further analyzed in [11] and termed polychronization. Since spike synchrony occurs with a temporal lag in this framework, one expects that, although ASSET is not designed to capture this type of coordinated spiking activity, it may still detect signatures of such activity by choosing a correspondingly larger bin width. We aim to explore such a scenario and other applications of ASSET to data from different types of network simulations that exhibit correlations on a fine temporal scale to study the potential of our method in identifying features of the underlying network model.

Importantly, increasing the number of neurons to be analyzed does not increase the computational cost of the method. Indeed, in order to evaluate significance values, the method relies on expressions involving a mere sum of the firing rates of individual neurons, which are virtually instantaneous to evaluate. Rapid advances in electrophysiology will soon enable the simultaneous recording of thousands of neurons [49]. These data promise to expose concerted mechanisms of neuronal coding that remained invisible so far. Our method is designed to keep up with these advances, and to be applicable to the next generation of large-scale recordings of spike data.

If an SSE occurs more than two times, it generates multiple DSs in the intersection matrix, each corresponding to one pair of occurrences. In [32] it was suggested to compute the overlap between triplets (or *n*-tuples) of bins rather than pairs, and generate a corresponding *n*-dimensional intersection matrix to visualize DSs in three (or *n*) dimensions. It is possible to extend this approach to ASSET and to exploit this higher-order evidence to increase the power of the method. This extension will be considered in future work.

Finally, we demonstrated the efficacy of ASSET on data of large-scale simulations of a balanced random network with embedded synfire chains, which were previously generated and analyzed in [12] with the original visual method. ASSET fully reconstructed the synfire chains active in the considered time period and did not return additional FPs. Differently from the original technique in [12], SSEs were here determined on the basis of their statistical significance and the results were obtained by an automated analysis workflow.

When analyzing real data, some parameters of the analysis such as the statistical thresholds *α*_1_ and *α*_2_ should be chosen optimally to minimize the risk of FPs, while at the same time not being excessively strict and thus missing true SSEs. Optimal values for the statistical thresholds can be inferred from independent surrogates of the original data, which can be created by one of several approaches, such as spike train shifting or spike dithering [50]. Such surrogate data contain slightly displaced spikes as compared to the original data, such that correlations in the original data (and in particular SSEs) are intentionally destroyed while other features of the data (e.g. firing rates or inter-spike interval regularities) are preserved. These uncorrelated data can be used to determine the expected value of entries in the probability and joint probability matrices under independence, and therefore to determine lower bounds for the thresholds *α*_1_ and *α*_2_ which ensure avoidance of FPs. Taking the least conservative of such values (the lower bounds) also minimizes the risk for FNs. Suitable values for other analysis parameters can be determined analogously. As illustrated in “Methods”, some of these parameters are tightly associated to putative features of the searched SSEs (for instance, the kernel length and kernel width to the temporal span and the wiggliness of the SSE, respectively), and may therefore be tuned in order to optimize the detection of SSE swith specific characteristics.

Taken together, these results demonstrate that ASSET is a reliable and effective tool to detect and identify repeated sequences of synchronous spiking activity in massively parallel spike train data. Whether synchrony propagation constitutes a mechanism of information processing in the neuronal circuitry still remains an open question, that belongs to the more general debate about the role of fine temporal coding versus rate coding. Convincing theoretical arguments as well as experimental evidence have been provided for both processing mechanisms (see e.g. [2, 6, 51] vs [26, 52, 53]). However, observing fine-scale temporal correlations requires the simultaneous observation and analysis of sufficiently large portions of the involved neuronal circuitry: the severe subsampling of such circuitry so far characterizing most available data prevents this analysis. Next-generation recording technology promises to expose this concerted activity, whose analysis may finally resolve the long-standing question about the role of millisecond-precise spike correlations in cognitive processing. Our work gives a contribution in this direction by providing, to the best of our knowledge, the first tool for a statistical analysis of synchrony propagation applicable to data of hundreds or thousands of simultaneously recorded neurons. The ASSET analysis method is available as part of the Electrophysiology Analysis Toolkit (Elephant; http://neuralensemble.org/elephant/). In future work we plan to employ the method to investigate the presence of SSE activity in electrophysiological recordings from awake animals and study SSE occurrence in relation to behavior.
